# Predicting the Length of Mechanical Ventilation in Acute Respiratory Disease Syndrome Using Machine Learning: The PIONEER Study

**DOI:** 10.3390/jcm13061811

**Published:** 2024-03-21

**Authors:** Jesús Villar, Jesús M. González-Martín, Cristina Fernández, Juan A. Soler, Alfonso Ambrós, Lidia Pita-García, Lorena Fernández, Carlos Ferrando, Blanca Arocas, Myriam González-Vaquero, José M. Añón, Elena González-Higueras, Dácil Parrilla, Anxela Vidal, M. Mar Fernández, Pedro Rodríguez-Suárez, Rosa L. Fernández, Estrella Gómez-Bentolila, Karen E. A. Burns, Tamas Szakmany, Ewout W. Steyerberg, the PredictION of Duration of mEchanical vEntilation in ARDS (PIONEER) Network

**Affiliations:** 1 CIBER de Enfermedades Respiratorias, Instituto de Salud Carlos III, 28029 Madrid, Spain; josu.estadistica@gmail.com (J.M.G.-M.); cafeoranestesia@gmail.com (C.F.); jmaelizalde@gmail.com (J.M.A.); prosu2001@yahoo.es (P.R.-S.); rosalidia.fernandez@gmail.com (R.L.F.); 2 Research Unit, Hospital Universitario Dr. Negrín, 35019 Las Palmas de Gran Canaria, Spain; cristina.fersan76@gmail.com (C.F.); estrellagbentolila@gmail.com (E.G.-B.); 3 Li Ka Shing Knowledge Institute at St. Michael’s Hospital, Toronto, ON M5B 1W8, Canada; Karen.Burns@unityhealth.to; 4 Faculty of Health Sciences, Universidad del Atlántico Medio (UNAM), 35017 Tafira Baja, Gran Canaria, Canary Islands, Spain; 5 Intensive Care Unit, Hospital Universitario Virgen de Arrixaca, 30120 Murcia, Spain; juasobar@hotmail.com; 6 Intensive Care Unit, Hospital General Universitario de Ciudad Real, 13005 Ciudad Real, Spain; alfonsoa@sescam.jccm.es; 7 Intensive Care Unit, Hospital Universitario de A Coruña, 15006 La Coruña, Spain; lidia.pita.garcia@sergas.es; 8 Intensive Care Unit, Hospital Universitario Río Hortega, 47012 Valladolid, Spain; mlfernandezrod@saludcastillayleon.es; 9 Surgical Intensive Care Unit, Department Anesthesia, Hospital Clinic, IDIBAPS, 08036 Barcelona, Spain; 10 Department of Anesthesia, Hospital Clínico Universitario de Valencia, 46010 Valenci, Spain; blancaarocas@hotmail.com; 11 Intensive Care Unit, Complejo Asistencial Universitario de León, 24001 León, Spain; mgonzalezvaq@saludcastillayleon.es; 12 Intensive Care Unit, Hospital Universitario La Paz, IdiPaz, 28046 Madrid, Spain; 13 Intensive Care Unit, Hospital Virgen de La Luz, 16002 Cuenca, Spain; elena03gon@gmail.com; 14 Intensive Care Unit, Hospital Universitario NS de Candelaria, 38010 Santa Cruz de Tenerife, Spain; gudru74@yahoo.es; 15 Intensive Care Unit, Hospital Universitario Fundación Jiménez Díaz, 28040 Madrid, Spain; anxelavidal@gmail.com; 16 Intensive Care Unit, Hospital Universitario Mutua Terrassa, 08221 Terrassa, Spain; marufaes@yahoo.es; 17 Thoracic Surgery, Hospital Universitario Dr. Negrín, 35019 Las Palmas de Gran Canaria, Spain; 18 Critical Care Medicine, Unity Health Toronto-St. Michael’s Hospital, Toronto, ON M5B 1W8, Canada; 19 Health Research Methods, Evidence, and Impact, McMaster University, Hamilton, ON L8S 4L8, Canada; 20 Intensive Care, Cardiff University, Cardiff CF14 4XW, UK; 21 Department Biomedical Data Sciences, Leiden University Medical Center, 2333 ZA Leiden, The Netherlands; E.W.Steyerberg@lumc.nl

**Keywords:** acute respiratory distress syndrome, lung-protective ventilation, duration of mechanical ventilation, machine learning, prediction models, observational studies, clinical trials

## Abstract

**Background**: The ability to predict a long duration of mechanical ventilation (MV) by clinicians is very limited. We assessed the value of machine learning (ML) for early prediction of the duration of MV > 14 days in patients with moderate-to-severe acute respiratory distress syndrome (ARDS). **Methods**: This is a development, testing, and external validation study using data from 1173 patients on MV ≥ 3 days with moderate-to-severe ARDS. We first developed and tested prediction models in 920 ARDS patients using relevant features captured at the time of moderate/severe ARDS diagnosis, at 24 h and 72 h after diagnosis with logistic regression, and Multilayer Perceptron, Support Vector Machine, and Random Forest ML techniques. For external validation, we used an independent cohort of 253 patients on MV ≥ 3 days with moderate/severe ARDS. **Results**: A total of 441 patients (48%) from the derivation cohort (n = 920) and 100 patients (40%) from the validation cohort (n = 253) were mechanically ventilated for >14 days [median 14 days (IQR 8–25) vs. 13 days (IQR 7–21), respectively]. The best early prediction model was obtained with data collected at 72 h after moderate/severe ARDS diagnosis. Multilayer Perceptron risk modeling identified major prognostic factors for the duration of MV > 14 days, including PaO_2_/FiO_2_, PaCO_2_, pH, and positive end-expiratory pressure. Predictions of the duration of MV > 14 days showed modest discrimination [AUC 0.71 (95%CI 0.65–0.76)]. **Conclusions**: Prolonged MV duration in moderate/severe ARDS patients remains difficult to predict early even with ML techniques such as Multilayer Perceptron and using data at 72 h of diagnosis. More research is needed to identify markers for predicting the length of MV. This study was registered on 14 August 2023 at ClinicalTrials.gov (NCT NCT05993377).

## 1. Introduction

Acute respiratory distress syndrome (ARDS) is a type of acute hypoxemic respiratory failure associated with lung injury and impaired gas exchange [1,2]. Mechanical ventilation (MV) is vital for most patients with moderate-to-severe ARDS managed in intensive care units (ICUs), although MV for long periods of time may induce lung injuries and infection [3,4]. The number of MV days is a major driver of high healthcare costs in managing critically ill patients [5]. Hence, a decrease in MV duration is an actionable research goal in ARDS management. The successful prediction of MV duration may impact several clinical decisions, such as the initiation of oral feeding, timing of performing a tracheostomy, transferring to long-term ventilation facilities, alignment with patients’ goals of care, or enrollment into clinical trials [6,7,8].

The prediction of the length of MV is important [6,7,9]. The mortality rate of patients requiring prolonged MV is high [9]. The accuracy of clinicians in predicting MV duration is very limited [6]. Usually, clinicians integrate multiple clinical features that are not well elaborated and make implicit assessments about the possible duration of MV, which are translated into routine medical practice with a wide margin of error [6]. Using logistic regression models, predictions of the duration of MV provide moderate levels of overall accuracy in critically ill patients and are insufficient to assist in clinical decisions [7,9,10]. Patients and clinicians require clinical prediction models to guide healthcare decisions in an evidence-based and personalized manner.

MV duration in ARDS is dependent on both ICU factors and patient-related factors. Advanced machine learning (ML) methods hold promise for enhancing accuracy in predicting MV duration [11,12]. ML is an exploratory process in which algorithms extract knowledge from the data provided. ML flexibility detects relationships between potential clinical features, physiologic parameters, and an outcome [13]. Despite extensive modeling and a large number of clinically relevant features, the discrimination using ML approaches for predicting responders to the prone position in mechanically ventilated patients with COVID-19 [14] was very poor. Scarce studies have evaluated the role of ML in predicting the duration of MV in ARDS patients [11,15,16,17]. Our primary goal was to compare the performance of logistic regression and three powerful ML approaches for the development, testing, and external validation of a model to predict the duration of MV > 14 days after diagnosis of moderate/severe ARDS. We hereto used a relatively large population of patients with moderate/severe ARDS under three different scenarios over time.

## 2. Methods

Approval of this study was permitted by the Ethics Committee at Hospital Universitario Dr. Negrin, Spain (CEI/CEIm #2021-321-1). Informed consent was waived based on Royal Decrees 1090/2015 and 957/2020 under the Spanish legislation for biomedical research, based on the retrospective nature of this comprehensive analysis, the anonymization/dissociation of data, and no potential harm or benefit to the patients. This study respected the TRIPOD (Transparent Reporting of a Multivariable Prediction Model for Individual Prognosis or Diagnosis) guidelines for reporting prediction models [18].

### 2.1. Patient Population and Study Design

This study is an extension of the Spanish SIESTA program [19,20] (Supplementary Materials). We performed a comprehensive analysis, termed the “PredictION of the duration of mEchanical vEntilation in aRds” (PIONEER) study on an unrestricted dataset from 1303 adult (>17 years) patients with moderate/severe ARDS [21] managed with lung-protective MV in a network of Spanish ICUs (Supplemental Materials). None of our ARDS patients had COVID-19. This study was performed in three steps. For the first two steps (model development and testing), we analyzed 1000 patients included in three prospective, multicenter, observational studies (see Supplemental Materials), admitting consecutive patients meeting the definition of moderate/severe ARDS [21]. In the third step, we tested the model performance in an independent cohort of moderate/severe ARDS patients [20] for reliable external validation [22,23]. 

For the purpose of this study and to avoid selection bias, we only analyzed patients with data from the first 3 ICU days on MV after the diagnosis of moderate/severe ARDS: data captured at the time of diagnosis (T0), data captured at 24 h (T24), and data captured at 72 h (T72). As a result, we excluded patients on MV < 3 days as follows: 80 patients from 1000 patients were included in the development/testing cohort, 50 patients from 303 patients were included in the validation cohort and finally analyzed data from 1173 patients. T0 was defined as the time and day in which the patient first met moderate/severe ARDS criteria, irrespective of the day of ICU admission or the initiation of MV, as mandated by the Berlin definition [21]. All patients had arterial blood gases at the study’s inclusion (Supplementary Materials). At T24, the values of gas-exchange and lung mechanics variables [including PaO_2_, PaCO_2_, PaO_2_/FiO_2_, and inspiratory plateau pressure (Pplat), among others] were evaluated under standardized ventilator settings [positive end-expiratory pressure (PEEP) of 10 cm H_2_O and FiO_2_ of 0.5)] [20]. When patients required PEEP > 10 or FiO_2_ > 0.5 and could not tolerate a reduction in PEEP or FiO_2_, the rules for setting PEEP and FiO_2_ were applied during the standardized evaluation, as validated by our group [24,25]. At other times, PEEP and FiO_2_ were set at the discretion of attending clinicians. We did not collect data from day 2. For T72, we used representative data at 72 h after the diagnosis of moderate/severe ARDS. We only included patients with moderate/severe ARDS. We excluded patients < 18 years old with acute heart failure, severe chronic pulmonary disease, do-not-resuscitate orders, brain death, or patients on MV for <24 h (see Supplementary Materials).

### 2.2. Variables and Outcomes

The selection of potentially clinically relevant variables was based on prior studies [25,26,27,28]. We included demographics, comorbidities, ventilator settings, lung mechanics [respiratory rate (RR), tidal volume (VT) as mL/kg predicted body weight (PBW), PEEP, Pplat, driving pressure (calculated as Pplat minus PEEP)] and gas-exchange (FiO_2_, PaO_2_, PaCO_2_, PaO_2_/FiO_2_, pH) at T0, T24, and T72. We also recorded the Acute Physiology and Chronic Health Evaluation II (APACHE II) [29] score during the first 24 h of ARDS diagnosis, the Sequential Organ Failure Assessment (SOFA) score [30], and the prevalence of extrapulmonary organ failures (OFs) included in the SOFA scale (Supplementary Materials). In each patient, we recorded data from 165 variables during their ICU stay (Supplementary Materials). We collected the date and status (alive/dead) of patients in the ICU and at hospital discharge.

We examined the performance of each method at T0, T24, and T72. For the purpose of our study, prolonged MV was defined as being ventilated for more than 14 days after the diagnosis of moderate/severe ARDS [6], independently of the number of patients who died between day 3 and day 14. 

### 2.3. Predefined Rules and Statistical Analysis

We focused on variables collected during the first 3 MV days after the diagnosis of moderate/severe ARDS to estimate the probability of MV duration > 14 days, independent of the cause of death or the underlying disease (Figure S1 and Table S1). For variable selection, our aim was to identify clinically relevant variables while avoiding redundant variables. We first analyzed the following features as potential predictors of prolonged MV: age, sex, comorbidities, SOFA score, number of extrapulmonary organ failures, PaO_2_, PaO_2_/FiO_2_, PaCO_2_, pH, FiO_2_, VT, RR, PEEP, Pplat, and driving pressure. To avoid multicollinearity [31], we performed matrices at T0, T24, and T72 to assess the correlation between variables in the dataset [32,33]. We also performed a principal component analysis to summarize the information content that could be easily visualized and analyzed [33,34] (Figures S3 and S4, Supplementary Materials).

We specified an a priori statistical analysis plan (Supplementary Materials). We identified potential variables that could be considered in the prediction model based on predefined rules and the contribution to the area under the receiver operating characteristic curve (AUC) in relation to MV duration. We assessed differences in the values of clinically relevant features at T0, T24, and T72 and across the development/testing cohort and the external validation cohort. We reported the odds ratio and 95% confidence intervals (CIs) and standard classification metrics (sensitivity, specificity, false positives, and false negatives) for each model. 

#### Feature Selection Method

Since the inclusion of all available variables in ML can lead to complex models that are difficult to interpret, we screened variables employing a genetic algorithm (GA) variable selection method [34] to achieve parsimony and identify a subset of relevant variables while excluding noise/redundant variables. GA variable selection is a technique that helps to identify a subset of the measured variables that are, for a given problem, the most useful for a precise and accurate regression model. Although many variables may be of use in prediction, several considerations may preclude measuring all the variables originally considered for a prediction model. It is useful to identify a subset of variables that allow sufficient prediction accuracy and precision while minimizing the number of variables to be measured. GAs provide a straightforward method based on a “survival of the fittest” approach to modeling data. GAs create random populations of artificial individuals that are evaluated by a mathematical fitness function and have been successfully applied to solve optimization problems, both for continuous and discrete functions (more details in Supplementary Materials). Our findings indicate that, for the purpose of our study, the GA approach is appropriate for finding an efficient subset of variables for combinations that are optimal for solving high-dimensional classification problems. The duration of MV can be treated as a classification problem. The selection of an optimized set of variables in our three early scenarios (T0, T24, T72) is key to the PIONEER study for predicting the prolonged duration of MV, especially when the search is large, complex or poorly understood, as in the setting of moderate/severe ARDS. We applied GA to optimize the subset of selected variables by reducing the Akaike information criterion (AIC) and the Bayesian information criterion (BIC) [35]. Lower values of both criteria are preferable. 

We performed statistical analysis using R (version 4.3.1, R Foundation for Statistical Computing, Vienna, Austria). A two-sided *p* < 0.005 was considered a real effect size [36]. 

We built the PIONEER prediction model by considering the lowest number of variables obtained by GA and providing the best performance of the three scenarios in the development database. We optimized the quality of models based on a 5-fold cross-validation approach for randomly splitting the development/training dataset and repeated this process 100 times (Supplementary Materials). We assessed the final lowest number of variables model using logistic regression and three supervised ML algorithms, Random Forest, Support Vector Machine, and Multilayer Perceptron [37,38], to generate prediction models for MV duration > 14 days after moderate/severe ARDS diagnosis. We also assessed the validity of the prediction models according to calibration and discrimination in an external validation cohort [39,40] (Supplementary Materials). 

**Figure 1:**
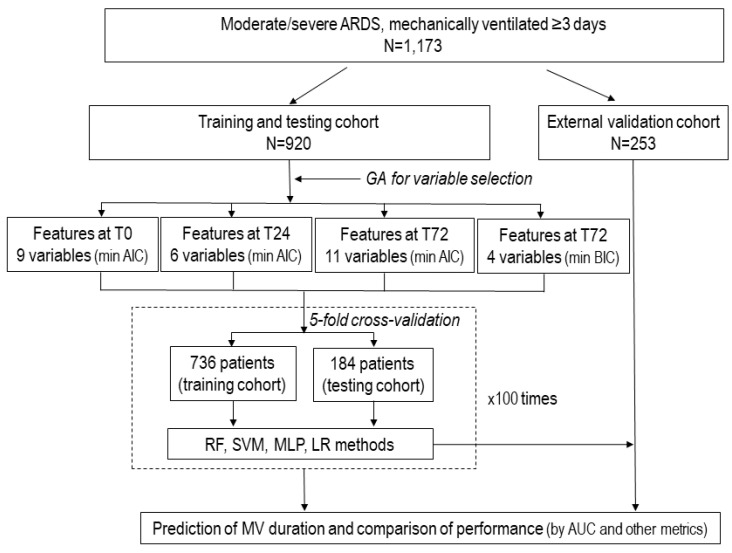
**Flow chart representing the study design of the PIONEER study.** The diagram illustrates the scheme for the database with 1173 patients with moderate-to-severe acute respiratory distress syndrome (ARDS), the selection of variables for final analysis, machine learning approaches, and comparison among the prediction models. Once the most relevant variables were selected by genetic algorithms in the dataset of 920 patients, and the model with the best performance (the highest area of the receiver characteristic curve, AUC) was identified, this dataset was divided into five folders to perform 5-fold randomized cross-validation repeated 100 times using machine learning techniques. Abbreviations: AUC: area under the receiver operating characteristic curve; GA: genetic algorithm: LR, logistic regression; MLP, Multilayer Perceptron; RF, Random Forest; SVM, Support Vector Machine; T0, time zero (at the time of diagnosis of moderate/severe ARDS; T24, at 24 h after diagnosis under standardized ventilator settings; and T72, at 72 h after diagnosis.

Figure 1 and Figure S2 summarize the study design.

## 3. Results

The baseline demographics, etiology, degree of severity, and outcome data of our patient population are reported in Table S2. The most common etiologies were pneumonia, sepsis, aspiration, and trauma in both cohorts (development and external validation). Most patients had moderate ARDS based on stratification by the Berlin criteria at T0. A total of 1173 patients [920 patients from the derivation cohort and 253 patients from the validation cohort] (Table 1) received MV for ≥3 days after moderate/severe ARDS diagnosis (Supplementary File), and their ICU mortality rate was similar (307/920, 33% vs. 77/253, 30%, respectively, *p* = 0.420) (Table S3). A total of 49% (441/920) patients from the derivation cohort and 40% (100/253) patients from the validation cohort were mechanically ventilated for >14 days [median 14 days (IQR 8–25) vs. 13 days (IQR 7–21), respectively]. A similar ICU mortality rate was found in patients ventilated for 3–14 days and for >14 days [155/479 (32%) vs. 152/441 (35%)] in the derivation cohort (Table 2 and Table S3).

From twenty clinically relevant variables collected at T0, T24, and T72 (Table S1), four variables were excluded for multicollinearity as follows: the PaO_2_, FiO_2_, SOFA score, and driving pressure. (Figure S3 and Tables S4–S6, Supplementary Results). Few characteristics remained associated with the duration of MV > 14 days in multivariable logistic regression analysis. The performance of the model with all 16 clinical variables had a cross-validated AUC of 0.61 at T0, 0.61 at T24, and 0.66 at T72. Principal component analysis showed that patients differed more at T72 than at T0 or T24 (Figure S4). When applying GA for variable selection and optimizing AIC and BIC (for both criteria, lower values are preferred), the resulting models reduced the number of predictors from 16 to 9, 6, 11, and 4 variables, respectively (Tables S7–S10). 

Among the variables with higher importance for predicting the duration of MV > 14 days at T72 were PaO_2_/FiO_2_, PaCO_2_, pH, and PEEP (Figure S5). The performance of ML methods and logistic regression for the scenarios in the development database is shown in Table 3. Multilayer Perceptron (MLP) provided the highest AUC values, and data collected at T72 provided better performance than data at T0 or T24.

Starting from 11 variables in the PIONEER model, the MLP technique was most promising for predicting MV duration > 14 days using data at T72 with the MLP technique (AUC 0.71, 95%CI 0.65–0.76). Models developed at one time period were not transferable to other time periods. The external validation of the best prediction models at T72 obtained from the derivation cohort for the duration of MV prediction is reported in Table S11. The ML models had poor calibration, implying the poor reliability of absolute risk predictions (Figure S6).

## 4. Discussion

In our study, the MLP technique predicted prolonged MV duration in patients with moderate-to-severe ARDS with modest accuracy. As expected, data captured at the baseline or at T24 were less predictive than data obtained at T72. Our findings highlight 4 out of 11 clinical features collected at T72 (Table S9) as follows: PaO_2_/FiO_2_, PaCO_2_, pH, and PEEP. Although these variables have been recognized as risk factors of prolonged MV, our study illustrates that absolute risk prediction based on these variables remains limited. 

The PIONEER prediction model could contribute to addressing some of these risk features for reducing the duration of MV in moderate/severe ARDS. We recognize that MV duration in ARDS is also dependent on ICU factors. Our study confirms that it is difficult to predict the time of ending MV in moderate/severe ARDS, even with modern ML techniques. Evolving clinical practice has led to the clinical use of non-invasive ventilation in mild and moderate ARDS [1,41]. Furthermore, organizational culture, staffing pressure, and various quality improvement initiatives implementing evidence-based management could be more important than patient-related factors. Our multicenter study cannot answer these issues since we would need data on all ICU-related factors, which are contextual, variable, and probably do not exist in an interpretable format. Given the available data, patients who would benefit the most from “actionable” timely therapeutic interventions or the early warning of possible adverse events [42] are those with the highest risk for prolonged MV duration [43]. The current ARDS framework [2] limits our ability to fully determine which patients need MV for >14 days. We do not know whether updating the ARDS definition addresses any of the existing limitations related to other aspects impacting ARDS recognition and management. A more refined prediction model that captures high-risk patients and ICU-related features in a timely manner could be beneficial. This would have the potential to guide clinicians in therapeutic choices in specific ARDS groups.

The early assessment of MV duration > 14 days may be important for evidence-based interventions that accelerate MV discontinuation [44]. Our findings suggest that MV duration only can be modestly predicted as early as T72. The selection of patients with a high risk of prolonged MV might affect significant clinical decisions, including referral to other centers, transfers to long-term ventilator units, the timing of performing a tracheostomy [8] or alignment with goals of care [6]. In addition, an early prediction model for the prolonged duration of MV could optimize ICU resource use [45]. Our model could determine with modest accuracy if a patient remains intubated after 14 days of ARDS diagnosis using commonly accessible variables during the first three MV days after diagnosis. In general, the requisite for a successful ML application is the presence of “useful patterns” in the data. In the current environment, it is far from optimized (if not unrealistic) to enable ML to provide a precise early prediction of prolonged MV duration in ARDS for clinicians. A comparison with previous ML studies on MV duration is difficult because investigators used different ML techniques, different timeframes, different ML metrics, or different populations [5,9,23,46]. Since the definition of prolonged MV is inconsistent, the performance assessment of related prediction models is not applicable to all situations [47]. We believe that using input criteria that maximize the measurement of variables commonly available to clinicians and minimize subjective clinician judgement is a preferable strategy. 

Clinicians are interested in actionable and modifiable variables for improving outcomes. It seems unrealistic that ventilated moderate/severe ARDS patients can be weaned quickly. We think that the following targets are reasonable within the first three days and beyond ARDS management to decrease MV duration as follows: improving oxygenation for PaO_2_/FiO_2_ > 150 mmHg [48], the use of high PEEP [49] while keeping Pplat < 29 cmH_2_O [50], and aiming to reduce extrapulmonary organ dysfunction [51]. Of note, the best strategies to reduce MV duration in patients with ARDS have not been identified yet and require further research. 

Of note, there is divergence in the use of invasive MV across countries [52], and patient characteristics vary markedly. This divergence highlights the need to better understand patient-, clinician-, and system-level choices associated with the use of MV. The impact of higher PEEP levels on ARDS has long been debated [53]. Available evidence suggests that high PEEP could be beneficial for moderate/severe ARDS. In the ALIVE epidemiological study [54], ICU mortality increased as the PaO_2_/FiO_2_ ratio was <150 mmHg and a pH ≤ 7.30. Permissive hypercapnia is sporadically required to allow lung-protective MV. After the induction of hypercapnia, pH decreases markedly and gradually moves toward normalization at 72 h. The clinical effects of hypercapnia are conflicting since metabolic acid-base adaptation triggered by hypercapnia is a complex process. pH compensation makes the VT reduction more acceptable in ARDS; however, concurrent infections could influence the metabolic adaptation to hypercapnia [55]. In a recent systematic review [56], permissive hypercapnia was associated with increased survival, although hypercapnia was imposed under lung-protective MV, and it was associated with a worse outcome. Changes in pH, PaCO_2_ or the PaO_2_/FiO_2_ ratio are strong predictors for disease progression in ARDS [57]. PaCO_2_ value is required for the calculation of alveolar dead space. We did not include dead space in the PIONEER model (end-tidal CO_2_ was not regularly measured in our patients throughout the first three days of MV), but we acknowledge that temporal variations in dead space during MV are associated with ICU outcomes [58] in addition to the early time-course of gas exchange in ARDS [59].

The strengths of our study include the fact that we analyzed a large number of patients from a multicenter network, reflecting the current clinical practice and contemporary case mix in moderate/severe ARDS. Starting from prior clinical knowledge represents an important strength leading to clinically relevant interpretations that are less likely to generate unreasonable predictions [60]. The use of general practices in ARDS across ICUs provides comparative data to support conclusions about the PIONEER model, allowing the potential utility to improve timely clinical interventions for ARDS. On the other hand, we also acknowledge some limitations of our study. First, we only investigated patients from the Spanish healthcare system managed with lung-protective MV; hence, the data might not be valid in other settings or in patients ventilated with large VT. Second, we ignored whether the model worked for each etiology, although they were manifested by the same syndrome. We felt that minimal exclusion criteria and a relatively large sample size attenuated these concerns. Third, our data were collected before the COVID-19 pandemic, and it is plausible that ARDS ventilator management might have changed since then [1]. Fourth, we trained our algorithms using only three ML techniques; others might lead to better prediction models. Fifth, we did not collect precise information on factors associated with weaning difficulties, such as sedatives, neuromuscular blocking agents, fluid balance, the level of consciousness, secondary infections, and muscle function. Sixth, our definition of prolonged MV excluded those who died before 14 days. Finally, the ML methods provided poorly calibrated predictions, implying that absolute risk predictions are not reliable for individual patients.

## 5. Conclusions

We confirmed that predicting MV duration in ARDS is complex, even with data captured at T72 compared to the baseline or at T24. ML techniques are promising but insufficient to provide reliable patient-level predictions. Clinical determinants of MV duration in ARDS are multifactorial and should be studied further to support the timely management and treatment of ARDS, focusing on optimizing gas exchange, lung strain, and variations in pH secondary to the natural evolution of the disease process. More research is needed to identify clinical and organizational variables that might help to recognize patients who are likely to have prolonged MV.

## Figures and Tables

**Table 1:** Descriptive characteristics of 1173 patients with moderate/severe ARDS at the time of diagnosis (T0) at 24 h (T24) and 72 h (T72) after diagnosis of moderate/severe ARDS.

Variable	Development Cohort			Testing Cohort		
	T0 n = 920	T24 n = 920	T72 n = 920	T0 n = 253	T24 n = 253	T72 n = 253
SOFA score	8.9 ± 3.3	8.9 ± 3.5	7.9 ± 3.9	9.7 ± 3.5	9.4 ± 3.9	8.3 ± 4.4
FiO_2_	0.79 ± 0.19	0.65 ± 0.17	0.63 ± 0.20	0.76 ± 0.20	0.62 ± 0.16	0.57 ± 0.19
PaO_2_, mmHg	85.9 ± 25.8	91.8 ± 26.8	108.1 ± 35.2	86.6 ± 25.3	98.3 ± 30.5	107.6 ± 32.9
PaO_2_/FiO_2_, mmHg	114.4 ± 37.5	150.2 ± 57.3	196.0 ± 81.7	120.8 ± 40.3	170.0 ± 65.9	221.5 ± 84.6
PaCO_2_, mmHg	49.1 ± 12.5	46.6 ± 10.1	48.1 ± 9.5	50.3 ± 13.7	47.1 ± 9.9	47.2 ± 9.2
pH	7.31 ± 0.11	7.35 ± 0.08	7.40 ± 0.08	7.30 ± 0.11	7.35 ± 0.08	7.40 ± 0.07
VT, ml/kg/PBW	6.9 ± 1.0	6.8 ± 0.9	6.9 ± 1.1	6.7 ± 1.1	6.5 ± 1.1	6.6 ± 1.2
Respiratory rate	21.4 ± 4.9	23.3 ± 5.0	24.7 ± 5.2	22.3 ± 4.4	23.4 ± 4.8	24.2 ± 4.9
PEEP, cmH_2_O	12.1 ± 3.3	12.5 ± 3.0	11.5 ± 3.3	11.1 ± 3.0	11.8 ± 2.8	10.7 ± 3.0
Pplat, cmH_2_O	26.4 ± 4.9	26.6 ± 4.6	24.5 ± 4.7	25.0 ± 4.9	24.7 ± 4.5	22.4 ± 4.4
Driving pressure, cmH_2_O	14.4 ± 4.9	14.2 ± 4.5	13.0 ± 4.4	14.0 ± 4.5	12.9 ± 4.2	11.6 ± 3.8
No. extrapulmonary OF	1.6 ± 1.1	1.7 ± 1.1	1.5 ± 1.2	1.9 ± 1.1	1.9 ± 1.2	1.6 ± 1.3

ARDS: acute respiratory distress syndrome, OF: organ failures, PBW: predicted body weight, PEEP: positive end-expiratory pressure, Pplat: inspiratory plateau pressure, SOFA: sequential organ failure assessment, T0: time of diagnosis of moderate/severe ARDS, T24: at 24 h of moderate/severe ARDS diagnosis, T72: at 72 h of moderate/severe ARDS diagnosis, and VT: tidal volume.

**Table 2:** Comparison of clinically relevant variables at the baseline (T0) in 920 patients of the development cohort in relation to the duration of mechanical ventilation from the time of diagnosis of moderate/severe ARDS.

Variable	MV 3–14 Days n = 479	MV > 14 Days n = 441	Mean Difference (95%CI)	*p*
Age, yr, mean ± SD	56.0 ± 16.8	57.1 ± 15.0	1.1 (−1 to 3.2)	0.292 *
Sex, n (%)				0.050 ^¶^
Male	311 (64.9)	313 (71.0)	6.1 (0.1 to 12.0)	
Female	168 (35.1)	128 (29.0)	6.1 (0.1 to 12.0)	
Etiology, n (%)				0.124 ^¶^
Pneumonia	226 (47.2)	232 (52.6)	5.4 (−1.6 to 11.8)	
Sepsis	142 (29.6)	104 (23.6)	6.0 (0.3 to 11.6)	
Aspiration	51 (10.6)	39 (8.8)	1.8 (−2.1 to 5.6)	
Trauma	37 (7.7)	35 (7.9)	0.2 (−3.3 to 3.8)	
Others	23 (4.8)	31 (7.0)	2.2 (−0.9 to 5.4)	
Degree of severity, n (%)				0.195 ^¶^
Severe	183 (38.2)	187 (42.4)	4.2 (−2.1 to 10.5)	
Moderate	296 (61.8)	254 (57.6)	4.2 (−2.1 to 10.5)	
APACHE II score	20.2 ± 6.6	20.6 ± 6.1	0.4 (−0.4 to 1.2)	0.352 *
SOFA score	8.7 ± 3.5	9.1 ± 3.1	0.4 (−0.01 to 0.8)	0.055 *
FiO_2_	0.79 ± 0.19	0.79 ± 0.19	0 (0 to 0)	1.0 *
PaO_2_, mmHg	87.8 ± 26.8	83.7 ± 24.5	−4.1 (−7.5 to −0.8)	0.016 *
PaO_2_/FiO_2_, mmHg	117.3 ± 38.4	111.4 ± 36.3	−5.9 (−10.7 to −1.1)	0.017 *
PaCO_2_, mmHg	48.4 ± 12.5	49.8 ± 12.4	1.4 (−0.1 to 3.1)	0.074 *
pH	7.31 ± 0.11	7.31 ± 0.10	0.0 (−0.01 to 0.01)	1.0 *
VT, ml/Kg PBW	6.9 ± 1.0	6.9 ± 1.0	0.0 (−0.1 to 0.1)	0.770 *
Respiratory rate, cycles/min	21.1 ± 4.9	21.7 ± 4.9	0.6 (−0.1 to 1.2)	0.093 *
PEEP, cmH_2_O	11.8 ± 3.3	12.3 ± 3.4	0.5 (0.03 to 0.9)	0.036 *
Plateau pressure, cmH_2_O	26.2 ± 5.0	26.6 ± 4.7	0.4 (−0.1 to 1.1)	0.119 *
Driving pressure, cmH_2_O	14.4 ± 4.9	14.4 ± 4.9	0 (−1.0 to 1.0)	0.852 *
No. extrapulmonary OF	1.5 ± 1.1	1.7 ± 1.0	0.2 (0.01 to 0.29)	0.030 *
All-cause ICU mortality, n (%)	155 (32.4)	152 (34.5)	2.1 (−4.0 to 8.2)	0.500 ^¶^

APACHE: acute physiology and chronic health evaluation, ARDS: acute respiratory distress syndrome, MV: mechanical ventilation, OF: organ failures, PBW: predicted body weight, PEEP: positive end-expiratory pressure, SD: standard deviation, SOFA: sequential organ failure assessment, VT: tidal volume. (*) Student’s *t*-test; and (^¶^) Fisher’s exact test.

**Table 3:** Comparison of analysis of performance using Random Forest, Support Vector Machine, Multilayer Perceptron, and logistic regression of the optimum model at T0, T24, and T72 in 920 patients with moderate/severe ARDS.

Time	Methods	Model	AUC (95%CI)	Sensitivity	Specificity	Accuracy	PPV	NPV
T0 (minimizing AIC)	Multilayer Perceptron	9-variable	0.66 (0.60–0.72)	0.60	0.63	0.62	0.60	0.63
	Random Forest	9-variable	0.54 (0.47–0.60)	0.46	0.60	0.53	0.51	0.55
	Support Vector Machine	9-variable	0.51 (0.43–0.59)	0.37	0.66	0.52	0.50	0.53
	Logistic regression	9-variable	0.58 (0.52–0.64)	0.63	0.55	0.59	0.58	0.64
T24 (minimizing AIC)	Multilayer Perceptron	6-variable	0.63 (0.56–0.69)	0.76	0.44	0.59	0.56	0.67
	Random Forest	6-variable	0.54 (0.47–0.61)	0.47	0.59	0.53	0.51	0.55
	Support Vector Machine	6-variable	0.51 (0.43–0.59)	0.40	0.64	0.52	0.50	0.54
	Logistic regression	6-variable	0.58 (0.52–0.64)	0.66	0.52	0.59	0.57	0.64
T72 (minimizing AIC)	Multilayer Perceptron	11-variable	0.71 (0.65–0.76)	0.53	0.74	0.64	0.65	0.63
	Random Forest	11-variable	0.61 (0.55–0.68)	0.46	0.68	0.57	0.57	0.58
	Support Vector Machine	11-variable	0.55 (0.40–0.65)	0.24	0.84	0.55	0.60	0.55
	Logistic regression	11-variable	0.63 (0.57–0.69)	0.64	0.61	0.62	0.61	0.66
T72 (minimizing BIC)	Multilayer Perceptron	4-variable	0.63 (0.57–0.70)	0.49	0.69	0.60	0.60	0.60
	Random Forest	4-variable	0.58 (0.50–0.65)	0.48	0.62	0.55	0.54	0.57
	Support Vector Machine	4-variable	0.53 (0.41–0.63)	0.50	0.58	0.54	0.52	0.56
	Logistic regression	4-variable	0.62 (0.56–0.68)	0.65	0.58	0.61	0.60	0.66

AIC: Akaike information criterion, AUC: area under the receiver operating characteristic curve, BIC: Bayesian information criterion, CI: confidence intervals, NPV: negative predictive value, and PPV: positive predictive value.

## Data Availability

All data needed to evaluate the conclusions in this article are presented and tabulated in the main text or the Supplemental Materials. Data are available from the corresponding author on reasonable request.

## Supplementary Materials

The following supporting information can be downloaded at: https://www.mdpi.com/article/10.3390/jcm13061811/s1.
