# Supplementary material for: Predicting the Length of Mechanical Ventilation in Acute Respiratory Disease Syndrome Using Machine Learning: The PIONEER Study
Source: J Clin Med. 2024 Mar 21;13(6):1811. doi: 10.3390/jcm13061811 (PMC10971349; doi:10.3390/jcm13061811)
Supplement: Supplementary file 1 [file jcm-13-01811-s001.zip › jcm-2879336-supplementary.pdf]

*SUPPLEMENTAL FILE**

Predicting length of mechanical ventilation in ARDS using machine learning: The PIONEER Study

Jesús Villar, **Jesús M. González-Martin**, **Cristina Fernández**, **Juan A. Soler**, **Alfonso Ambrós**, **Lidia Pita-García**, **Lorena Fernández**, **Carlos Ferrando**, **Blanca Arocas**, **Myriam González-Vaquero**, **José M. Añón**, **Elena González-Higueras**, **Dácil Parrilla**, **Ánxela Vidal**, **M. Mar Fernández**, **Pedro Rodríguez-Suárez**, **Rosa L. Fernández**, **Estrella Gómez-Bentolila**, **Karen E.A. Burns**, **Tamas Szakmany**, **Ewout W. Steyerberg**,

*For the PredictION of duration of mEchanical vEntilation in ARDS (PIONEER) Network**

*This Supplemental File has been provided by the authors for additional information about their work.

Corresponding author:
Jesús Villar, MD, PhD
Multidisciplinary Organ Dysfunction Evaluation Research Network
Research Unit
Hospital Universitario Dr. Negrín
Barranco de la Ballena s/n, 4th Floor – South wing.
35019 Las Palmas de Gran Canaria, Spain.
Email: jesus.villar54@gmail.com

The PIONEER Study

TABLE OF CONTENTS

**SUPPLEMENTARY METHODS**	page 3
Ethical approval	page 3
Patient population	page 3
Study design	page 4
General care	page 6
Variables and outcome	page 7
Statistical analysis plan	page 8
Predefined rules, pre-specified statistical analysis, & variable selection	page 8
Building the development and validation datasets	page 11
External validation	page 12
Data analysis	page 12
**SUPPLEMENTARY RESULTS**	page 14
TABLES S1 to S11	page 15 to page 25
FIGURES S1 to S6	page 26 to page 31
**SUPPLEMENTARY REFERENCES**	page 32
**APPENDIX 1. Centers and Members of the PIONEER Project**	page 38
**APPENDIX 2. Members and Centers of the SIESTA Network**	page 39

SUPPLEMENTARY METHODS

This comprehensive analysis was an investigator-initiated study from multicenter, clinical observational studies conducted in a network of intensive care units (ICUs) from several geographical areas of Spain.

The purpose of the study was to develop and validate an early prediction model for duration of mechanical ventilation (MV) longer than 14 days in patients with moderate-to-severe acute respiratory distress syndrome (ARDS) using machine learning (ML) techniques.

Ethics Approval

This study was approved by the Ethics Committee for Clinical Research at the Hospital Universitario Dr. Negrín, Las Palmas de Gran Canaria, Spain (Reference CEI/CEIm 2021-321-1). The need for informed consent was waived based on Spanish legislation for biomedical research (Royal Decree 1090/2015 December 2015, and Royal Decree 957/2020 November 2020) due to the retrospective nature of the secondary analysis, the anonymization/dissociation of data, and no potential for harm or benefit to patients.

This study was conducted in accordance with the principles of the Declaration of Helsinki approved by the World Medical Association [1], the Convention of the European Council related to human rights and biomedicine, the International Code of Medical Ethics of the World Medical Association [2], and within the requirements established by the Spanish legislation for biomedical research, the protection of personal data, and bioethics. None of the findings reported in the present study have been published elsewhere.

The study followed the Transparent Reporting of a multivariable prediction model for Individual Prognosis Or Diagnosis (TRIPOD) guidelines for prediction models [3].

Patient population

This study is an extension of the ***S***panish ***I***nitiative for ***E***pidemiology, ***S***tratification and ***T***herapies of ***A***cute Respiratory Distress Syndrome (SIESTA) Program [4-7] (*members are listed in Appendix 1 and Appendix 2 of this Supplemental File*). We performed a comprehensive analysis, termed the PIONEER ("Predict**I**ON of duration of mEchanical vEntilation in aRds") Study (registered on August 14th 2023 at ClinicalTrials.gov: NCT NCT05993377), of an unrestricted dataset derived from 1,303 adult (>17 years) patients with moderate-to-severe ARDS [8] treated with lung-protective MV in a network of ICUs from several geographical areas of Spain.

Study design

This study was conducted in three steps. In the first two steps (model development and testing), we analyzed data derived from 1,000 patients included in three independent, prospective, multicenter, observational, and non-interventional cohorts, enrolling consecutive patients meeting current criteria for moderate/severe ARDS [8]. In the ALIEN cohort [4], 22 participating ICUs included 300 patients from September 2008 to May 2010 (NCT00736892). In the STANDARDS cohort [9,10], 24 participating ICUs included 300 patients from September 2013 to July 2015 (NCT02288949). The STANDARDS-2 cohort [11], 21 participating ICUs admitted 400 patients from August 2015 to April 2017 (NCT02836444).

In the third step, we tested the performance of the model in an independent cohort of 303 patients with moderate/severe ARDS included in the multicenter observational PANDORA study [7]. Patients were admitted in a network of 22 ICUs from May 2017 to March 2018 (NCT03145974). With this approach, we studied the temporal aspect and external validity of predicting prolonged duration of MV days as primary endpoint in future observational studies and clinical trials, since the new cohort contains recently treated ARDS patients. The external validation cohort has sufficient number of events required for external validation [12], recommended by recent guidelines [13].

Patients admitted to participating ICUs were screened daily during the study periods. All patients were intubated and mechanically ventilated. All consecutive patients (in the ALIEN cohort) meeting the American-European Consensus Conference (AECC) criteria for ARDS [14] on positive end-expiratory pressure (PEEP) ≥5 cmH_2O, and the Berlin criteria for moderate or severe ARDS [8] (in the STANDARDS, STANDARDS-2, and PANDORA cohorts) were included for this analysis. Of note, by leaving the assessment of PaO_2/FiO_2 essentially unchanged, the AECC definition and the Berlin criteria are basically identical. The requirement of a minimum PEEP level of 5 cmH_2O has no impact on the definition since all patients with ARDS were managed with PEEP ≥5 cmH_2O. Our screening applies only to patients with moderate-to-severe ARDS, which include: (i) having an initiating clinical condition (pneumonia, aspiration, overdose, sepsis, trauma, acute pancreatitis, etc.), (ii) within one week of a known clinical insult or new or worsening respiratory symptoms, (iii) bilateral pulmonary infiltrates on chest imaging, (iv) absence of left atrial hypertension or no clinical signs of left heart failure, and (v) hypoxemia (as defined by a $PaO_2/FiO_2 \leq 100$ mmHg on PEEP≥5 cmH_2O for severe ARDS, and 100 mmHg<$PaO_2/FiO_2 \leq 200$ mmHg on PEEP≥5 cmH_2O for moderate ARDS, regardless of FiO_2). We only included patients with moderate/severe ARDS. We did not enroll patients with persistent mild ARDS

during the entire ICU stay. However, no patients with mild ARDS were excluded if they moved to a more severe category, although we do not have data on the precise number of those patients. We excluded patients <18 years old, with severe chronic pulmonary disease, acute heart failure, with a do-not-resuscitate orders, brain death, or patients receiving MV for <24 hours.

For the purpose of this study and to avoid selection bias, we only analyzed patients with MV data from the first three ICU days after diagnosis of moderate/severe ARDS: data captured at the time of diagnosis of moderate/severe ARDS (T0), at 24 hours (T24), and at 72 hours (T72). Data from day 2 were not collected in the parent studies. Therefore, we only considered patients on MV for ≥3 days with data at T0, T24, and at T72 to compare patients with data at those time-periods. As a result, we excluded 130 patients on MV for <3 days (80 from 1,000 patients included in the development/testing cohort and 50 from the 303 patients included in the validation cohort), and finally analyzed data from 920 patients in the derivation/testing cohort, and 253 patients from the external validation cohort.

T0 was defined as the day in which the patient first met moderate/severe ARDS criteria, irrespective of the day of ICU admission or initiation of MV, as mandated by the Berlin definition [8]. All patients had arterial blood gases at study inclusion. We did not use peripheral capillary oxygen saturation (SpO_2) as a surrogate for PaO_2 for enrolling patients. At T24, values of gas-exchange and lung mechanics [including PaO_2, $PaCO_2$, PaO_2/FiO_2, inspiratory plateau pressure (Pplat), among others] were assessed in all patients under standardized ventilator settings [positive end-expiratory pressure (PEEP) of 10 cmH_2O and FiO_2 of 0.5] [7]. When patients required PEEP>10 or FiO_2>0.5 and could not tolerate a decrease in PEEP or FiO_2, a set of rules for setting PEEP and FiO_2 were applied *only* during the standardized assessment, as described and validated previously by our group [11,15]. At other times, PEEP and FiO_2 levels were set at the discretion of managing clinicians. For T72, we used representative data at 72 hours after diagnosis of moderate/severe ARDS.

For appropriate identification of patients with moderate/severe ARDS, attending physicians considered qualifying blood gases only while patients were clinically stable, and did not consider transient falls in PaO_2 resulting from acute events unrelated to the disease process (such as obstruction of endotracheal tube by secretions, endotracheal suctioning, ventilator disconnection, or sudden pneumothorax). Also, because diagnostic inclusion could occur with other diseases that cause hypoxemia and have bilateral pulmonary infiltrates on radiographs, clinicians excluded lymphangitic

carcinomatosis, acute eosinophilic pneumonia, hypersensitivity pneumonitis, and idiopathic pulmonary fibrosis [16].

General Care

Attending clinicians followed current guidelines for general critical care management, which included the following: (i) in case of sepsis, physicians were urged to ensure early identification of causative microorganism, intravenous administration of antibiotics as soon as bacterial sepsis was suspected or recognized, and to optimize antibiotic selection and timely administration on the bases of antibiogram; (ii) fluid resuscitation and vasopressor use were individualized with the goal of maintaining a systolic blood pressure ≥90 mmHg or a mean arterial pressure ≥65 mmHg; (iii) to maintain hemoglobin between 7-10 g/dL. For ventilatory management, clinicians followed current recommendations for lung-protective ventilation with a tidal volume (VT) of 4-8 mL/kg predicted body weight (PBW), a Pplat <30 cmH_2O, a ventilatory rate (RR) to maintain a $PaCO_2$ between 35-50 mmHg (permissive hypercapnia was allowed to target VT), and PEEP and FiO_2 combinations according to the PEEP-FiO_2 table of the ARDSnet protocol [17], ensuring that among the PEEP and FiO_2 combinations, clinicians should use the PEEP levels that allowed the reduction of FiO_2 to the lowest levels for maintaining a PaO_2 within a target range of 60 to 100 mmHg or a SpO_2 within a target range of 90 to 98%. Routine blood parameters were left to the discretion of the responsible physician, but it was not mandatory to record them, although clinicians used routinely that information for diagnosis and magamen5t of the underlying disease process, for scoring organ dysfunction, and as mandated by SOF scale and for APACHE II score.

The choice of drugs for sedation and analgesia, early neuromuscular blockade, prone positioning, recruitment maneuvers, hemodynamic management modalities, and the decision to perform a tracheostomy were left to the discretion of the attending physician. PBW was calculated using the following formula: 50 + 0.91 x [height (cm) – 152] for men, and 45.5 + 0.91 x [height (cm) – 152] for women [17]. Although prone positioning and recruitment maneuvers were used in some patients, we do not have data on timing of prone positioning, or whether prone ventilation and recruitment maneuvers were applied as a rescue therapy, as a routine practice, or following any specific protocol. Features highly dependent on ICU variability, such as active treatment and chronic conditions reporting, were not recorded to improve usability across health systems.

Weaning off MV was not strictly protocolized, but could be started when the attending physician considered it clinically appropriate. Patients were assessed daily for readiness for a spontaneous

breathing trial (SBT) based on the ARDSnet protocol [17]. In general, prerequisites for the SBT included a partial reversal of the underlying cause of ARDS, a PaO_2/FiO_2 >200 mmHg with PEEP <10 cmH_2O and FiO_2 ≤0.4, no vasopressors, continuous sedation minimized, and ability to cough during tracheal aspirations. Spontaneous ventilation was tested with a T-piece or with pressure support at 8 cmH_2O. The duration of the SBT was at least 30 min and no longer than 120 min. If the patient passed the trial, a decision for extubation was taken, unless there was a specific reason not to extubate. Weaning and the decision to extubate were left to the discretion of the responsible physician. Since the rate of reintubation after extubation for all indications is estimated at about 20% [18], patients at high risk for reintubation [>65 years of age, or hypercapnic ($PaCO_2$ >45 mmHg after extubation), ineffective cough and excessive secretions, ≥1 weaning failure, with more than one comorbid condition, upper airway obstruction, or APACHE II score >12 on the day of extubation], non-invasive ventilatory support for 24 to 48 hours was indicated until stable or requiring reintubation [19].

Variables and Outcomes

Data were collected in each participating ICU using standardized case report forms and transmitted to the coordinating center (Hospital Universitario Dr. Negrin) when the patient was discharged from hospital. Before exporting the data into a computerized database, a trained data collector from the coordinating center checked the completeness and the quality of information. Logical checks were performed for missing data and for finding inconsistences, especially regarding clinical diagnosis, dates, and severity scores. If necessary, the data collector contacted the investigator to validate the data or reformat the data for entry into the database.

Selection of clinically relevant variables was based on our previous studies [11,20]. To build the models, we analyzed information from 165 variables including demographics, comorbidities, and data from ventilator settings and lung mechanics [VT, RR, PEEP, Pplat, driving pressure (calculated as Pplat minus PEEP) and gas exchange (PaO_2, $PaCO_2$, FiO_2, PaO_2/FiO_2, pH)] at T0, T24 and T72. Attending physicians recorded the most common comorbidities (we only considered comorbidities with a prevalence >5%), as reported in our previous work [20]: neoplastic diseases, liver disease, cardiac disease, immunosuppression, diabetes, and morbid obesity. Neoplastic diseases included cancer in solid organs and hematological malignancies. Being immunosuppressed or immunocompromised was a result of certain diseases or conditions, or because of medication or treatment for a disease or condition, including, but not limited to cancer or organ transplantation. We also recorded the APACHE

II (Acute Physiology And Chronic Health Evaluation II) score [21] during the first 24 hours of ARDS diagnosis, the Sequential Organ Failure Assessment (SOFA) score [22], and the occurrence of extrapulmonary organ system failures included in the SOFA scale (cardiovascular system, liver, kidney, coagulation system, and central nervous system). Since the term “organ dysfunction” is unclear and because organ dysfunction may emerge from reasons other than sepsis, extrapulmonary organ failure was defined as an acute change in organ-specific SOFA score ≥2 [23,24]. The baseline SOFA score was assumed to be zero in patients not known to have preexisting organ dysfunction. Sepsis was defined according to 2001 International Consensus Conference criteria [25]. We recorded the actual duration of MV, the length of stay in the ICU, and date and status (alive or dead) at ICU and hospital discharge.

Our primary goal was to compare the performance of three machine learning (ML) methods and conventional statistics in predicting prolonged duration of MV after the diagnosis of moderate/severe ARDS over time. We examined the performance of each method with respect to T0, T24, and T72. For the purpose of this study, prolonged MV was defined as being ventilated for >14 days after diagnosis of moderate/severe ARDS, based on previous work by our group [26].

Statistical analysis plan

Predefined rules, pre-specified statistical analysis, and variable selection

We defined and specified in advance rules and expectations before the final statistical and machine learning analysis was conducted, realizing that overly detailed analysis could produce overoptimistic results due to a combination of reduced statistical power to detect real differences, or due to an increase in the variance around the mean estimates, and/or an increased statistical likelihood of a false finding when too many variables are examined.

Since feature selection is important in building a prediction model that is easily actionable and interpretable in clinical decision making, we collected data from 165 variables in each patient during their ICU stay. We focused on variables collected at T0, T24 and T72 to estimate the probability of duration of MV>14 days, independent of the underlying disease or whether the patient died (**Figure S1**) based on previous work by our group [9-11]. Our aim for variable selection was to incorporate clinically relevant variables while avoiding noise/redundant variables.

We *first* described the full dataset of patients with moderate-to-severe ARDS ventilated for at least three days (n=920). Thus, we listed the values of each variable for all 920 patients at T0, T24, and

T72. The distribution of values for all variables identified a wide range of duration of MV. *Second*, we initially considered the following features as potential predictors of prolonged MV: age, sex, comorbidities (neoplastic diseases, liver disease, cardiac disease, immunosuppression, diabetes, morbid obesity), SOFA score, number of extrapulmonary OF, PaO_2, PaO_2/FiO_2, $PaCO_2$, pH, FiO_2, VT, RR, PEEP, Pplat, and driving pressure. We did not include respiratory compliance in the model because it shares collinearity with three Independent variables needed for its calculation (VT, Pplat, and PEEP). According to the panel of experts of the Berlin definition [8], respiratory compliance did not contribute to the predictive validity of severe ARDS for mortality and it was removed from the ARDS definition. Although we have the APACHE II score [21] reported at T0 and T24, we did not include it in the model because it is a cumbersome score designed for the first 24 hours of ICU admission, made of 12 physiological variables and two disease-related variables, it is not routinely calculated at the bedside in most ICUs worldwide or during trial enrollment decisions, it requires numerous data elements, and relies on laboratory data that are not uniformly collected. In addition, at least half of the variables needed to calculate the APACHE II score are included in the list of selected variables, such as age, PaO_2, FiO_2, respiratory rate, pH, renal function, neurological function, and comorbidities.

Third, we identified potential variables that could be included in the prediction models based on our redefined rules and their contribution to the area under the receiver operating characteristic (ROC) curve (AUC), and their p-values in relation to duration of MV (**Table S1**). A ROC curve essentially has two components represented by the sensitivity and 1-specificity [27]. AUC is an effective way to summarize the overall prognostic accuracy of a variable or test, and it is most useful for assessing relevance of treatment effects. When representing and reporting the AUC, the point corresponding to no change (AUC=0.5) is represented by a diagonal line (45-degree line or no discriminatory ability for the outcome of interest).

Fourth, although several variables could share collinearity with other independent variables (FiO_2 and PaO_2 for the calculation of the PaO_2/FiO_2 ratio; Pplat and PEEP for the calculation of driving pressure), we considered all variables at the initial steps of our analysis. Whether driving pressure relates causally to outcome remains to be established in randomized controlled trials, despite we valued Pplat in our previous studies [10,11,28]. Other variables seemed to have redundancy (the value of SOFA score and the number of extrapulmonary OF). To avoid multicollinearity, we built correlation matrices at T0, T24, and T72 as a statistical tool to calculate the linear relationship between two variables in the

dataset [29-31] for excluding features with multicollinearity (**Figure S3**). The matrix shows how all possible pairs of values in a table that are related to each other. It is a powerful tool for summarizing a large data set and showing patterns in the data. The correlation coefficient ranges from -1 to +1, where 1 is considered a strong positive relationship between variables, 0 means a neutral relationship, and -1 means a negative correlation. We also performed a principal component analysis (PCA) as a statistical procedure that allows summarizing the information content by means of a smaller set of "summary indices" that can be more easily visualized and analyzed [31-33] (**Figure S4**). It is a popular multivariate statistical technique used in pattern recognition and signal processing based on projection methods. The goal of PCA is to extract the important information from the data and to express this information as a set of summary indices called principal components [31-33]. The first and second principal component provide an approximation of what model or projection is better. The first principal component represents the maximum variance direction in the data. The second principal component reflects the second largest source of variation in the data. When the two principal components are derived, they define a plane. In the specific context of our study, adding the numerical values of both components provides a tendency in the direction of data in favor of which day seems better positioned to predict MV>14 days.

Fifth, variable selection or feature subset selection is a common task in machine learning (ML) or data mining models. ML is a branch of artificial intelligence (AI) encompassing two major approaches: supervised and unsupervised learning [34]. The objective of supervised ML is to develop an algorithm capable of predicting a unique output when provided with a specific input. The expectations are that the resulting algorithm would deliver accurate predictions when exposed to new and never before data. Since the inclusion of all available variables in a ML model could produce noisy results which are difficult to interpret, we screen variables employing a genetic algorithm (GA) variable selection method [35] as a technique to achieve parsimony and to identify a subset of relevant and significant variables (subset selection) for a potential accurate prediction model, while excluding noise/redundant variables. GA variable selection is a technique that helps to identify a subset of the measured variables that are, for a given problem, the most useful for a precise and accurate regression model. Although many variables may be of use in a prediction, several considerations may preclude measuring all the variables originally considered for a prediction model. In these cases, it is useful to identify a subset of variables that allow sufficient prediction accuracy and precision while minimizing the number of variables to be measured. GA provide a straightforward method based on a "survival of the fittest" approach to modelling data. GA

is a heuristic search algorithm mimicking the process of biological evolution and natural selection [36]. GA creates random populations of artificial individuals that are evaluated by a mathematical fitness function. GAs have been successfully applied to solve optimization problems, both for continuous and discrete functions. In this sense, variable selection for logistic regression models can be regarded as an optimization problem, and thus can be solved by GAs [37,38]. Our findings indicate that for the purpose of our study, the GA approach is appropriate for finding an efficient subset of variables for combinations that are optimal for solving high dimensional classification problems. Duration of MV can be treated as a classification problem. The selection of an optimized set of variables in our three early scenarios (T0, T24, T72) is key in the PIONEER study for predicting prolonged duration of MV, especially when the search is large, complex or poorly understood, as in the setting of moderate/severe ARDS. We decided to use GA for feature/variable selection due to our previous successful experience with it [39].

Sixth, when applying GA for variable selection, we optimize the subset of selected variables by minimizing the Akaike information criterion (AIC) and the Bayesian information criterion (BIC) [40] (**Figure S2**). AIC is an estimator of prediction error and thereby relative quality of statistical models for a given set of data. BIC is a criterion for model selection among a finite set of models. For both criteria, lower values are preferred. We also calculated the variance inflation factor (VIF), a measure of multicollinearity in regression logistic analysis. Multicollinearity exists when there is a correlation between multiple independent variables in a multiple regression model. For the purpose of our study, only variables with a VIF<5 were included in the model [30]. We searched in the data for model specification since the model was not pre-specified. For building the PIONEER prediction model for moderate/severe ARDS patients, we considered the model with the minimum number of variables that provided a similar or better performance as the full-variable predictor model (**Tables S4-S10**).

Seventh, we evaluated the final model with the minimum number of variables at each time scenario using three supervised ML techniques: Random Forest (RF), Support Vector Machine (SVM) and Multilayer Perceptron (MLP) [41-45]. RF is a supervised ML algorithm, which combines the output of multiple decision trees to reach a single result. The RF algorithm is made up of a collection of decision trees, and each tree in the ensemble is comprised of a data sample drawn from a training set with replacement [43]. While decision trees consider all the possible feature split, RF only select a subset of those features, resulting in precise predictions. SVM is one of the prevailing algorithms because the data in biomedical research are often limited. One of the main strengths of the SVM is the ability to

efficiently construct complex decision boundaries from limited samples [41,44]. The deep learning MLP is a feed for forward neural network with a basic architecture comprising fully connected layers [44,45]. The input layer has the same number of inputs that the total predictor variables. The middle layer looks for characteristics associated with the data. The output layer had the same number of outputs as the categories to predict. The R packages for ML that we used are the following: Caret, pROC, randomForest, Keras, and e1071.

For ML techniques, input data were randomly split into training data (train), made up of 80% of the 920 records (736 rows), and the remaining 20% (184 rows) were used as test data. With the intention of reducing noise, each of the selected variables was normalized using the following formula:

$$Z = X – \min (X) / [\max (X) – \min (X)]$$

Where X is one of the selected genes, min (X) is the minimum value, max (X) is the maximum value, and Z is the resulting variable that was used for the ML process.

Building the development and validation datasets

A popular approach is to randomly split the data into two parts: one to develop the model (training cohort) and another to measure its performance (validation cohort). However, this split-sample method is often inefficient [46,47]. We used a 5-fold cross-validation for splitting randomly the 920-patient dataset as 736 patients for development (training) and 184 patients for testing (validating). This computerized technique replicates the process of sample generation by drawing samples with replacement from the original dataset [48], which means that the data set is divided into 5 folds, and in each run, 4 (80%) were used for training (n=736) and the remaining 1 (20%) was used for testing (n=184). We repeated the 5-fold cross-validation 100 times for obtaining a stable estimate with the mean values of 500 validation samplings.

Calculations were performed using the R Core Team software 2023 (R version 4.3.1) (https://www.r-project.org) (R Foundation for Statistical Computing, Vienna, Austria).

We compared the predictive performance of the three ML methods using the following parameters: accuracy, sensitivity, specificity, from the confusion matrix for both the validation and the

test dataset [49]. These values were calculated using the indicators true positive, false positive, false negative and true negative.

External validation

As the third step, and for solving the complexity of validation of our prediction model, we tested the performance of the model in fully new patients. We analyzed a cohort of 253 consecutive patients ventilated ≥3 days with moderate-to-severe ARDS included in the multicenter PANDORA study [7]. With this approach, we studied the temporal aspect of external validity of the model since this independent cohort contains recently treated patients. The external validation cohort had a sufficient number of events (≥100 patients ventilated >14 days) required for external validation [12]. As recommended by recent guidelines [13], we avoided the retraining on the external dataset.

Data analysis

We calculated the mean, standard deviation (SD), median and the interquartile range (IQR) of quantitative variables. We used the Kolmogorov-Smirnov test to examine the normal distribution of data. We calculated the frequency and percentage of qualitative variables. We reported data as percentages or mean ± SD, unless otherwise specified. We reported the odds ratio and 95% confidence intervals (CI). We assessed differences in the values of clinically relevant features in the three scenarios (T0, T24, T72), and across the development/testing cohort and the external validation cohort. We analyzed differences between distributions of categorical variables with the Fisher's exact test. We identified potential variables that could be included in the prediction model based on our predefined rules, the AUC, and their p-values. For all comparisons, a two-sided significance level of p-value <0.005 was considered a real effect size, as recommended [50].

We measured the mean decrease in Gini coefficient as a measure of how each variable contributes to the homogeneity in the resulting RF algorithm model [51]). We also calculated three measures (intercept, calibration slope, and *c*-statistic) to assess the validity of the prediction models, related to calibration and discrimination, and plotted graphically, by studying the external validity of the models developed in 920 patients and tested in 253 patients [47,52].

SUPPLEMENTARY RESULTS

ICU and hospital mortalities of development and validation cohorts were similar [307/920 (33.4%) vs. 77/253 (30.4%), p=0.420; and 347/920 (37.7% vs. 87/253 (34.4%, p=0.368, respectively] (**Table S2**). From the original derivation/testing cohort of 1000 patients, 80 patients (8%) were on MV for <3 days, and most of them (68/80, 85%) died in the ICU due mainly to multisystem organ failure (n=47, 69.1%), refractory sepsis/septic shock (n=10, 14.7%), and refractory hypoxemia (n=8, 11.8%). Only 12 patients out of 80 (15%) on invasive MV for <3 days from the derivation cohort survived at ICU discharge.

Considering ICU mortality in relation to duration of MV, ICU mortality rates of both subgroups (MV>14 days vs. MV 3-14 days) were similar in the derivation [152/441 (34.5%) vs. 155/479 (32.4%), OR 1.10 (95%CI 0.84-1.45) (p=0.529), respectively] and validation cohorts [25/100 (25.0%) vs. 52/153 (34.0%), OR 1.54 (95%CI 0.88-2.71) (p=0.162), respectively] (**Table S3**).

Median duration of MV in 920 patients from the derivation cohort was 14 days (IQR: 8-25 days): 441 patients (47.9%) were on MV>14 days and 479 (52.1%) were ventilated for 3-14 days. From 479 patients on MV for 3-14 days, almost a third of them (n=155, 32.4%) died in the ICU, mainly due to multisystem organ failure (n=68, 43.9%), refractory hypoxemia (n=38, 24.5%), and refractory sepsis/septic shock (n=24, 15.5%). In the validation cohort of 253 patients, where the median duration of MV was 13 days (IQR: 7-21 days): 100 patients (39.5%) were on MV>14 days and 153 (60.5%) were ventilated for 3-14 days.

Using a correlation matrix at T0, T24, and T72, we identified that PaO_2/FiO_2 was highly correlated with FiO_2 and PaO_2, Pplat with driving pressure, and SOFA score with the number of extrapulmonary OF (**Figure S3**). As a result, we eliminated from the analysis the following variables: PaO_2, FiO_2, SOFA score, and driving pressure. The principal component analysis (PCA) supported the finding that the two clusters (MV>14 days vs. MV 3-14 days) differed more at T72 than at baseline or at T24 (**Figure S4**).

The final model minimizing AIC and BIC for prediction of duration of MV>14 days was based on values of variables at T72. We found that PaO_2/FiO_2, $PaCO_2$, pH, and PEEP at T72 were among the most predictive features (**Figures S5, S6**), suggesting that most features collected at baseline or at 24h were irrelevant or useless for early prediction of duration of MV>14 days in patients with moderate/severe ARDS. Models developed at one time period are not transferable to other time periods.

Table S1. Univariate logistic regression of 20 clinically relevant variables in 920 patients with moderate-to-severe ARDS ventilated ≥3 days, in relation to prediction of duration of mechanical ventilation >14 days.

Variables	b	SE	OR (95%CI)	P-value	AUC
Age	0	0	1 (1.0 – 1.01)	0.29	0.52
Sex (male)	0.28	0.14	1.32 (1.0 – 1.75)	0.05	0.53
Cardiac disease	-0.35	0.26	0.71 (0.42 – 1.18)	0.186	0.51
Diabetes	-0.04	0.19	0.96 (0.66 – 1.38)	0.818	0.50
Immunosuppressed	0.27	0.22	1.3 (0.84 – 2.03)	0.234	0.51
Morbid obesity	0.38	0.23	1.47 (0.93 – 2.33)	0.098	0.52
Liver disease	-0.04	0.29	0.96 (0.54 – 1.71)	0.886	0.50
Neoplastic disease	0.06	0.17	1.06 (0.76 – 1.48)	0.730	0.50
SOFA at T0	0.04	0.02	1.04 (1.0 – 1.08)	0.055	0.55
SOFA at T24	0.05	0.02	1.05 (1.01 – 1.09)	0.011	0.56
SOFA at T72	0.05	0.02	1.05 (1.01 – 1.08)	0.007	0.57
VT at T0	0.02	0.06	1.02 (0.9 – 1.15)	0.791	0.50
VT at T24	-0.05	0.07	0.95 (0.82 – 1.1)	0.474	0.51
VT at T72	-0.04	0.06	0.96 (0.85 – 1.09)	0.527	0.51
FiO_2 at T0	0.16	0.35	1.17 (0.59 – 2.32)	0.649	0.51
FiO_2 at T24	0.73	0.38	2.07 (0.99 – 4.36)	0,055	0.54
FiO_2 at T72	1.33	0.34	3.79 (1.96 – 7.37)	<0.001	0.59
Respiratory rate at T0	0.02	0.01	1.02 (1.0 – 1.05)	0.093	0.54
Respiratory rate at T24	0.03	0.01	1.03 (1.0 – 1.06)	0.033	0.54
Respiratory rate at T72	0-04	0.01	1.04 (1.02 – 1.07)	0.001	0.56
PEEP at T0	0.04	0.02	1.04 (1.0 – 1.08)	0.037	0.54
PEEP at T24	0.04	0.02	1.05 (1.0 -1.09)	0.047	0.54
PEEP at T72	0.08	0.02	1.09 (1.04 – 1.13)	<0.001	0.58
Plateau pressure at T0	0.02	0.01	1.02 (0.99 – 1.05)	0.122	0.53
Plateau pressure at T24	0.03	0.01	1.03 (1.0 – 1.06)	0.033	0.54
Plateau pressure at T72	0.05	0.01	1.06 (1.03 – 1.09)	<0.001	0.58
Driving pressure at T0	0	0.01	1 (0.98 – 1.03)	0.844	0.51
Driving pressure at T24	0.01	0.01	1.01 (0.99 – 1.04)	0.32	0.52
Driving pressure at T72	0.02	0.01	1.02 (0.99 – 1.05)	0.309	0.53
PaO_2 at T0	-0.01	0	0.99 (0.99 – 1.0)	0.016	0.55
PaO_2 at T24	0	0	1 (0.99 – 1.0)	0.061	0.52
PaO_2 at T72	0	0	1 (0.99 – 1.0)	0.101	0.53
PaO_2/FiO_2 at T0	0	0	1 (0.99 – 1.0)	0.017	0.54
PaO_2/FiO_2 at T24	0	0	1 (0.99 -1.0)	0.012	0.55
PaO_2/FiO_2 at T72	0	0	1 (0.99 – 1.0)	<0.001	0.59
$PaCO_2$ at T0	0.01	0.01	1.01 (1.0 – 1.02)	0.074	0.54
$PaCO_2$ at T24	0-02	0.01	1.02 (1.01 – 1.04)	<0.001	0.57
$PaCO_2$ at T72	0.03	0.01	1.03 (1.02 – 1.05)	<0.001	0.59
pH at T0	-0.18	0.62	0.84 (0.25 – 2.85)	0.776	0.50
pH at T24	-1.82	0.8	0.16 (0.03 -0.78)	0.023	0.54
pH at T72	-0.04	0.84	0.96 (0.18 – 5.02)	0.958	0.51
No. extrapulmonary OF at T0	0.14	0.06	1.15 (1.01 – 1.3)	0.033	0.55
No. extrapulmonary OF at T24	0.14	0.06	1.15 (1.02 – 1.3)	0.019	0.55
No. extrapulmonary OF at T72	0.11	0.06	1.11 (1.0 – 1.24)	0.06	0.55

ARDS: acute respiratory distress syndrome, AUC: area under the receiver operating characteristic curve, b: beta, CI: confidence intervals, OF: organ failures, OR: odds ratio, PEEP: positive end-expiratory pressure, SE: standard error, SOFA: sequential organ failure assessment, T0: at the time of moderate/severe ARDS diagnosis, T24: at 24 hr after moderate/severe ARDS diagnosis, T72: at 72 hr after moderate/severe ARDS diagnosis, VT: tidal volume.

Table S2. Baseline demographics, etiology, degree of severity, and outcome data of 1,173 patients with moderate/severe ARDS.

**Variables**	**Development cohort** n = 920	**Testing cohort** n = 253	**p-value**
Age, yr, mean ± SD	56.5 ± 16.0	57.2 ± 14.7	0.531*
Sex, n (%)			0.043¶
Male	624 (67.8)	189 (74.7)	
Female	296 (32.2)	64 (25.3)	
Etiology, n (%)			
Pneumonia	458 (49.8)	92 (36.4)	<0.001¶
Sepsis	246 (26.7)	60 (23.7)	0.374¶
Aspiration	90 (9.8)	45 (17.8)	<0.001¶
Trauma	72 (7.8)	35 (13.8)	0.005¶
Acute pancreatitis	23 (2.5)	8 (3.2)	0.718¶
Multiple transfusions	9 (1.0)	3 (1.2)	0.729¶
Others	22 (2.4)	10 (4.0)	0.258¶
Degree of severity, n (%)			0.134¶
Severe	370 (40.2)	88 (34.8)	
Moderate	550 (59.8)	165 (65.2)	
APACHE II, mean ± SD	20.4 ± 6.4	20.6 ± 7.7	0.688*
Days on MV, mean ± SD	19.6 ± 17.8	16.0 ± 15.8	0.004*
All-cause ICU mortality, n (%)	307 (33.4)	77 (30.4)	0.420¶
All-cause hospital mortality, n (%)	347 (37.7)	87 (34.4)	0.368¶

APACHE: acute physiology and chronic health evaluation, ARDS: acute respiratory distress syndrome, ICU: intensive care unit, IQR: interquartile range, MV: mechanical ventilation.

(*) Student's t-test; (¶) Fisher's exact test

Table S3. Distribution and outcome of patients with moderate/severe ARDS in relation to duration of mechanical ventilation. ARDS: acute respiratory distress syndrome, CI: confidence intervals, ICU: intensive care unit, MV: mechanical ventilation, OR: odds ratio, T0: time of diagnosis of moderate/severe ARDS.

**Duration of MV**	**Derivation cohort** (N=920) n	ICU deaths	**Validation cohort** (N=253) n	ICU deaths	Difference (95%CI) in ICU mortality (derivation vs. validation)<br><br>OR (95%CI)	p-value
3-14 days from T0	479	155 (32.4%)	153	52 (34.0%)	1.6% (-6.7 to 10.4)<br><br>0.93 (0.63 to 1.37)	0.714<br><br>0.767
>14 days from T0	441	152 (34.5%)	100	25 (25.0%)	9.5% (-0.7 to 18.2)<br><br>1.58 (0.96 to 2.58)	0.068<br><br>0.077
Difference (95%CI) ICU deaths (3-14 vs. >14 days)<br><br>OR (95%CI)	2.1% (-4.0 to 8.2)<br><br>0.91 (0.69 to 1.20)		9.0% (-2.7 to 19.8)<br><br>1.54 (0.88 to 2.71)		-<br><br>-	-<br><br>-
p-value	0.500<br>0.529		0.129<br>0.162		-	-

Table S4. Performance of a model predicting duration of MV>14 days considering clinically relevant variables (n=16) collected at diagnosis (T0) in 920 patients with moderate/severe ARDS using logistic regression analysis. PaO_2, FiO_2, driving pressure, and SOFA (sequential organ failure assessment) score are not included due to multicollinearity (see text for details). Data are expressed as mean values of logistic coefficients.

Variables	b	SE	OR (95%CI)	p-value	VIF
Intercept	-14.66	6.47	0 (0 - 0.13)	0.024	
Age	0.01	0	1.01 (1 - 1.02)	0.093	1.17
Sex (male)	0.34	0.15	1.41 (1.05 - 1.89)	0.021	1.06
Cardiac disease	-0.51	0.27	0.6 (0.35 - 1.02)	0.062	1.08
Diabetes	-0.06	0.20	0.94 (0.64 – 1.39)	0.773	1.06
Immunosuppressed	0.32	0.24	1.37 (0.86 – 2.2)	0.184	1.09
Obesity	0.37	0.24	1.45 (0.9 – 2.35)	0.128	1.08
Liver disease	-0,19	0.31	0.83 (0.45 – 1.52)	0.547	1.09
Neoplastic disease	-0.05	0.18	0.96 (0.67 – 1.36)	0.798	1.08
VT	0.12	0.07	1.12 (0.97 – 1.3)	0.117	1.30
Respiratory rate	0.02	0.02	1.02 (0.99 – 1.05)	0.153	1.18
PEEP	0.03	0.02	1.03 (0.98 – 1.08)	0.220	1.27
Plateau pressure	0.01	0.02	1.01 (0.98 – 1.04)	0.687	1.18
PaO_2/FiO_2	0	0	1 (0.99 – 1.0)	0.052	1.08
$PaCO_2$	0.01	0.01	1.01 (1 – 1.03)	0.043	1.73
pH	1.59	0.85	4.89 (0.93 – 26.24)	0.062	1.78
No. extrapulmonary OF	0.16	0.07	1.17 (1.02 – 1.35)	0.021	1.17
AIC	1273.26				
BIC	1355.28				
AUC	0.6125				

ARDS: acute respiratory distress syndrome, AIC: Akaike information criterion, AUC: area under the receiver operating characteristic curve, b: beta, BIC: Bayesian information criterion, CI: confidence intervals, OF: organ failures, OR: odds ratio, PEEP: positive end-expiratory pressure, SE: standard error, VIF: variance inflation factor, VT: tidal volume.

Table S5. Performance of a model predicting duration of MV>14 days considering clinically relevant variables (n=16) collected at 24 hours of diagnosis (T24) of 920 patients with moderate/severe ARDS using logistic regression analysis. PaO_2, FiO_2, driving pressure, and SOFA (sequential organ failure assessment) score are not included due to multicollinearity (see text for details). Data are expressed as mean values of logistic coefficients.

Variables	b	SE	OR (95%CI)	p-value	VIF
Intercept	-6.7	7.93	0 (0 – 6778)	0.398	
Age	0.01	0	1.01 (1 - 1.02)	0.078	1.16
Sex (male)	0.3	015	1.34 (1.01 – 1.8)	0.046	1.05
Cardiac disease	-0.48	0.28	0.62 (0.36 – 1.05)	0.080	1.07
Diabetes	-0.06	0.2	0.94 (0.64 – 1.39)	0.757	1.07
Immunosuppressed	0.3	0.24	1.35 (0.85 – 2.17)	0.202	1.08
Obesity	0.38	0.24	1.47 (0.91 – 2.38)	0.118	1.08
Liver disease	-0.22	0.31	0.8 (0.43 – 1.48)	0.481	1.08
Neoplastic disease	-0.08	0.18	0.92 (0.65 – 1.32)	0.665	1.09
VT	0.05	0.08	1.05 (0.89 – 1.24)	0.549	1.20
Respiratory rate	0.02	0.01	1.02 (0.99 – 1.05)	0.247	1.21
PEEP	0.02	0.03	1.02 (0.97 – 1.07)	0.375	1.21
Plateau pressure	0.01	0.02	1.01 (0.97 – 1.04)	0.737	1.32
PaO_2/FiO_2	0	0	1 (1 – 1)	0.235	1.14
$PaCO_2$	0.02	0.01	1.02 (1 – 1.04)	0.029	1.65
pH	0.53	1.03	1.71 (0.23 – 12.95)	0.604	1.59
No. extrapulmonary OF	0.13	0.07	1.14 (1 – 1.31)	0.046	1.20
AIC	1273.40				
BIC	1355.46				
AUC	0.6119				

ARDS: acute respiratory distress syndrome, AIC: Akaike information criterion, AUC: area under the receiver operating characteristic curve, b: beta, BIC: Bayesian information criterion, CI: confidence intervals, OF: organ failures, OR: odds ratio, PEEP: positive end-expiratory pressure, SE: standard error, VIF: variance inflation factor, VT: tidal volume.

Table S6. Performance of a model predicting duration of MV>14 days considering clinically relevant variables (n=16) collected at 72 hours of diagnosis (T72) of 920 patients with moderate/severe ARDS using logistic regression analysis. PaO_2, FiO_2, driving pressure, and SOFA (sequential organ failure assessment) score are not included due to multicollinearity (see text for details). Data are expressed as mean values of logistic coefficients.

Variables	b	SE	OR (95%CI)	p-value	VIF
Intercept	-34.07	8.1	0 (0 - 0)	<0.001	
Age	0.01	0	1.01 (1 – 1.02)	0.071	1.16
Sex (male)	0.38	0.15	1.46 (1.09 – 1.97)	0.012	1.05
Cardiac disease	-0.52	0.28	0.59 (0.34 – 1.03)	0.066	1.07
Diabetes	0.01	0.2	1.01 (0.68 – 1.51)	0.948	1.07
Immunosuppressed	0.39	0.24	1.48 (0.92 – 2.4)	0.109	1.08
Obesity	0.32	0.25	1.38 (0.85 – 2.26)	0.198	1.08
Liver disease	-0.22	0.31	0.8 (0.43 – 1.47)	0.475	1.06
Neoplastic disease	0	01.8	1 (0.69 – 1.44)	0.996	1.10
VT	0.1	0.07	1.1 (0.96 – 1.27)	0.168	1.19
Respiratory rate	0.03	0.01	1.03 (1 – 1.06)	0.036	1.21
PEEP	0.05	0.02	1.05 (1.01 – 1.1)	0.029	1.28
Plateau pressure	0.01	0.02	1.01 (0.98 – 1.05)	0.410	1.33
PaO_2/FiO_2	0	0	1 (0.99 – 1)	<0.001	1.17
$PaCO_2$	0.03	0.01	1.03 (1.01 – 1.05)	0.002	1.38
pH	4.06	1.07	57.81 (7.22 - 483.9)	<0.001	1.48
No. extrapulmonary OF	0.13	0.07	1.14 (1.01 – 1.3)	0.040	1.23
AIC	1232.5				
BIC	1314.47				
AUC	0.6600				

ARDS: acute respiratory distress syndrome, AIC: Akaike information criterion, AUC: area under the receiver operating characteristic curve, b: beta, BIC: Bayesian information criterion, CI: confidence intervals, OF: organ failures, OR: odds ratio, PEEP: positive end-expiratory pressure, SE: standard error, VIF: variance inflation factor, VT: tidal volume.

Table S7. Performance of a model of predicting MV duration >14 days at the time of diagnosis (T0) of 920 patients with moderate/severe ARDS, using logistic regression analysis and minimizing the Akaike information criterion. The model reduced the number of selected variables from 16 to 9.

Variable	b	SE	OR (95%CI)	p-value	VIF
Intercept	-13.33	6.4	0 (0 – 0.42)	0.037	
Age	0.01	0	1.01 (1 – 1.02)	0.129	1.07
Sex (male)	0.3	0.15	1.36 (1.02 – 1.81)	0.038	1.03
Cardiac Disease	-0.53	0.27	0.59 (0.34 – 1)	0.052	1.07
Obesity	0.39	0.24	1.48 (0.93 – 2.37)	0.098	1.02
Respiratory rate	0.02	0.01	1.02 (0.99 – 1.05)	0.056	1.05
PaO_2/FiO_2	0	0	1 (0.99 – 1)	0.023	1.03
$PaCO_2$	0.01	0.01	1.01 (1 – 1.03)	0.052	1.64
pH	1.61	0.84	5.01 (0.97 – 26.46)	0.056	1.76
No. extrapulmonary OF	0.16	0.07	1.17 (1.03 – 1.34)	0.018	1.1
AIC	1265.36				
BIC	1313.61				
AUC	0.6055				

ARDS: acute respiratory distress syndrome, AIC: Akaike information criterion, AUC: area under the receiver operating characteristic curve, b: beta, BIC: Bayesian information criterion, CI: confidence intervals, OF: organ failures, OR: odds ratio, SE: standard error, VIF: variance inflation factor.

Table S8. Performance of a model of predicting MV duration >14 days at 24 hours (T24) after the diagnosis of 920 patients with moderate/severe ARDS, using logistic regression analysis and minimizing the Akaike information criterion. The model reduced the number of selected variables from 16 to 6.

Variable	b	SE	OR (95%CI)	p-value	VIF
Intercept	-1.86	0.43	0.16 (0.07 – 0.36)	<0.001	
Age	0.01	0	1.01 (1 – 1.02)	0.136	1.06
Sex (male)	0.27	0.15	1.31 (0.99 – 1.75	0.061	1.02
Cardiac Disease	-0.48	0.27	0.62 (0.36 – 1.06)	0.082	1.06
Obesity	0.42	0.24	1.53 (0.96 – 2.44)	0.073	1.02
$PaCO_2$	0.02	0.01	1.02 (1.01 – 1.04)	0.002	1.01
No. extrapulmonary OF	014	0.06	1.15 (1.02 – 1.3)	0.019	1.01
AIC	1260.70				
BIC	1294.52				
AUC	0.5974				

ARDS: acute respiratory distress syndrome, AIC: Akaike information criterion, AUC: area under the receiver operating characteristic curve, b: beta, BIC: Bayesian information criterion, CI: confidence intervals, OF: organ failures, OR: odds ratio, SE: standard error, VIF: variance inflation factor.

Table S9. Performance of a model of predicting MV duration >14 days at 72 hours (T72) after the diagnosis of 920 patients with moderate/severe ARDS, using logistic regression analysis and minimizing the Akaike information criterion. The model reduced the number of selected variables from 16 to 11.

**Variable**	**b**	**SE**	**OR (95%CI)**	**p-value**	**VIF**
Intercept	-33.36	8.13	0 (0 - 0)	<0.001	
Age	0.01	0	1.01 (1 – 1.02)	0.071	1.10
Sex (male)	0.36	0.15	1.43 (1.07 – 1.92)	0.018	1.04
Cardiac disease	-0.5	0.28	0.60 (0.34 -1.04)	0.074	1.07
Immunosuppressed	0.36	0.24	1.43 (0.9 – 2.29)	0.135	1.04
VT	0.1	0.07	1.11 (0.96 – 1.27)	0.152	1.19
Respiratory rate	0.03	0.01	1.03 (1.0 – 1.06)	0.027	1.19
PEEP	0.06	0.02	1.07 (1.02 – 1.11)	0.004	1.11
PaO_2/FiO_2	0	0	1.0 (0.99 – 1.0)	<0.001	1.11
$PaCO_2$	0.03	0.01	1.03 (1.01 – 1.05)	0.001	1.36
pH	3.99	1.06	54.21 (6.88 – 446)	<0.001	1.46
No. extrapulmonary OF	0.12	0.06	1.13 (1.0 – 1.28)	0.055	1.16
AIC	1225.30				
BIC	1283.23				
AUC	0.6582				

ARDS: acute respiratory distress syndrome, AIC: Akaike information criterion, AUC: area under the receiver operating characteristic curve, b: beta, BIC: Bayesian information criterion, CI: confidence intervals, OF: organ failures, OR: odds ratio, PEEP: positive end-expiratory pressure, SE: standard error, VIF: variance inflation factor.

Table S10. Performance of a model of predicting MV duration >14 days at 72 hours (T72) after the diagnosis of 920 patients with moderate/severe ARDS, using logistic regression analysis and minimizing the Bayesian information criterion (BIC). The model reduced the number of variables from 16 to 4.

**Variable**	**b**	**SE**	**OR (95%CI)**	**p-value**	**VIF**
Intercept	-21.64	7.36	0 (0 – 0)	0.003	
PEEP	0.06	0.02	1.06 (1.02 – 1.11)	0.004	1.07
PaO_2/FiO_2	0	0	1 (0.99 – 1)	<0.001	1.10
$PaCO_2$	0.03	0.01	1.03 (1.01 – 1.04)	0.001	1.24
pH	2.75	0.98	15.59 (2.32 – 108.02)	0.005	1.26
AIC	1232.50				
BIC	1256.67				
AUC	0.6313				

ARDS: acute respiratory distress syndrome, AIC: Akaike information criterion, AUC: area under the receiver operating characteristic curve, b: beta, BIC: Bayesian information criterion, CI: confidence intervals, OF: organ failures, OR: odds ratio, PEEP: positive end-expiratory pressure, SE: standard error, VIF: variance inflation factor.

Table S11. External validation of the 11 and 4-variable model at T72 in an independent cohort of 253 patients with moderate/severe ARDS. NOTE: We used the 920-patient population as the training cohort and the new 253 patients as the testing cohort.

Time	Methods	Model	AUC (95%CI)	Sensitivity	Specificity	Accuracy	PPV	NPV
T72	Multilayer Perceptron	11-variable	0.51 (0.44-0.58)	0.41	0.58	0.51	0.39	0.60
	Random Forest	11-variable	0.51 (0.44-0.58)	0.38	0.61	0.52	0.39	0.60
	Support Vector Machine	11-variable	0.45 (0.38-0.53)	0.19	0.80	0.56	0.38	0.60
	Logistic regression	11-variable	0.50 (0.42-0.57)	0.63	0.44	0.52	0.43	0.65
T72	Multilayer Perceptron	4-variable	0.57 (0.50-0.64)	0.21	0.82	0.58	0.43	0.61
	Random Forest	4-variable	0.54 (0.46-0.61)	0.45	0.59	0.54	0.42	0.62
	Support Vector Machine	4-variable	0.56 (0.48-0.63)	0.19	0.80	0.56	0.38	0.60
	Logistic regression	4-variable	0.52 (0.44-0.59)	0.48	0.59	0.55	0.44	0.64

AUC: area under the receiver operating characteristic curve, CI: confidence intervals, NPV: negative predictive value, PPV: positive predictive value.

Figure S1. Time line for the early prediction model on duration of mechanical ventilation (MV) >14 days. In many cases, the duration of MV finalized at the time of ICU death. The duration of MV was predicted using the data at the time of moderate/severe ARDS diagnosis, at 24 h, and at 72 hours later.

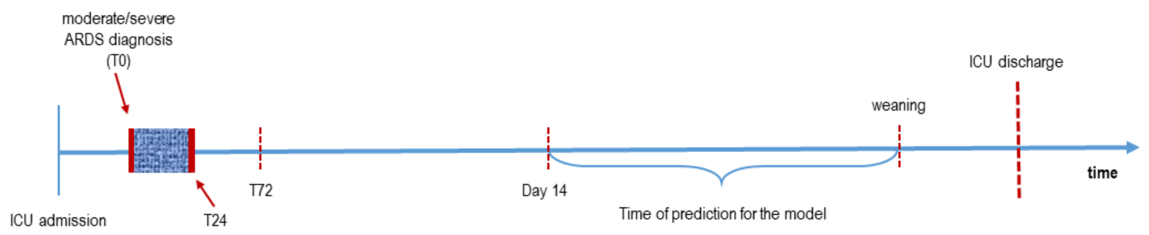

Figure S2. Analysis flowchart. *AIC: Akaike information criterion; ARDS: acute respiratory distress syndrome; BIC: Bayesian information criterion (see text for details).*

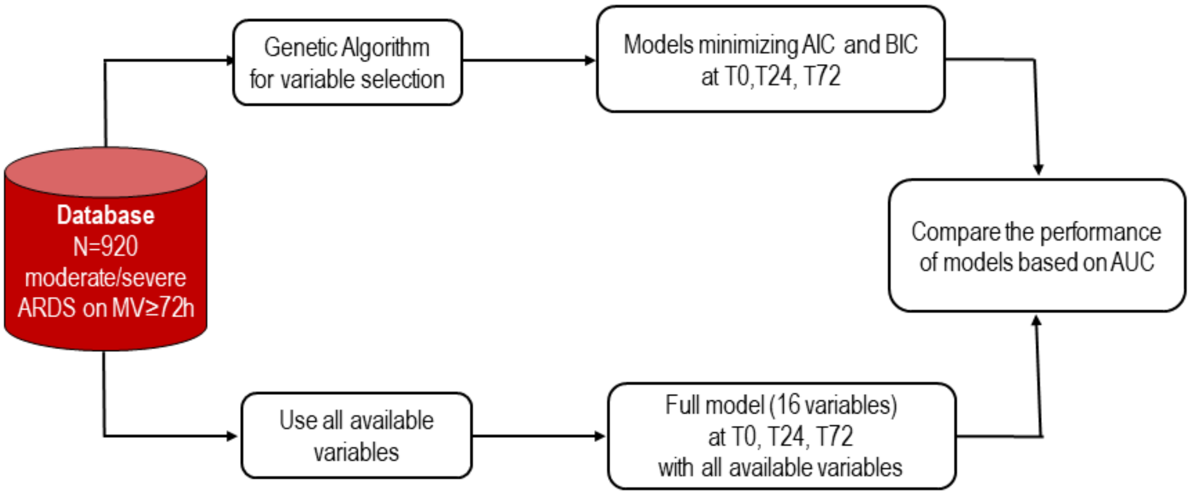

Figure S3: Correlation matrices at T0, T24 and T72 of 12 variables associated with MV duration. Blue represents positive correlation, and red represents negative correlation. The area of the pie chart represents the specific value of correlation coefficients.

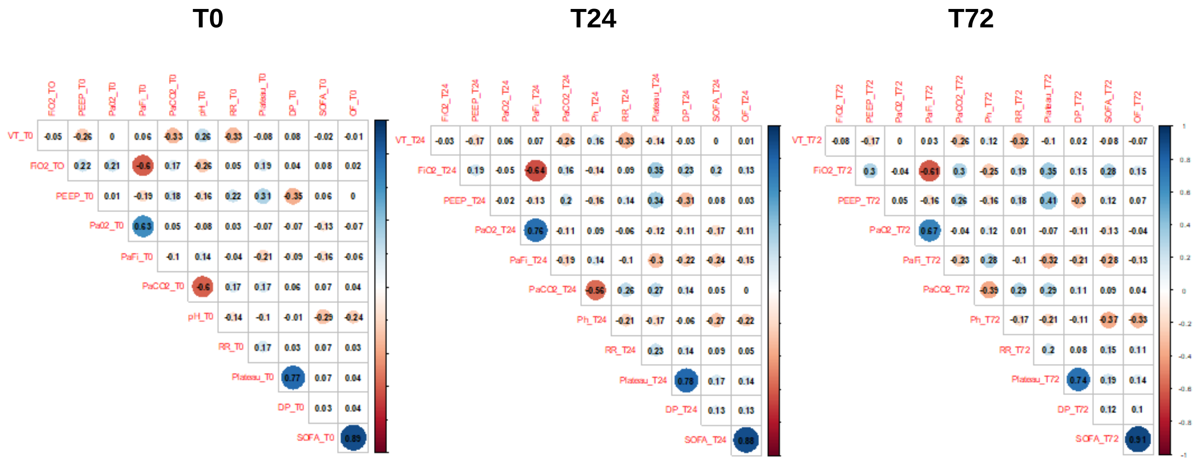

Figure S4. Principal component analysis (PCA) of predictors of MV duration in ARDS patients (Cluster of patients on MV>14 days is marked in blue and cluster of patients on MV ≤14days in red). Although there is overlapping between the clusters, the two clusters differed more at T72 (Dim1 + Dim2 = 43.7 + 21.8 = 65.5) than at any other study time.

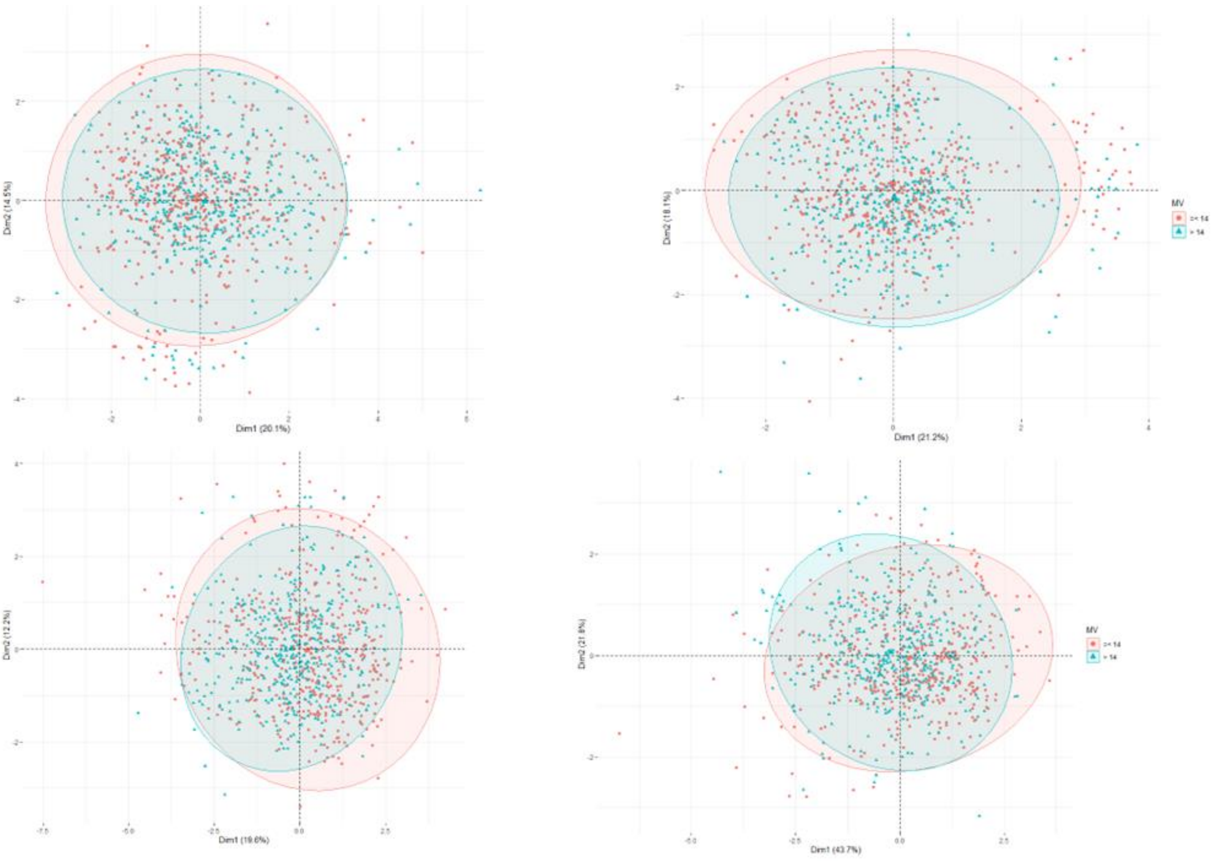

Figure S5. Variable importance of variables at T0, T24 and T72 of 9, 6, 11 and 4 variable models for prediction of duration of MV>14 days in terms of mean decrease in Gini using Random Forest classification. The mean decrease in GINI coefficient is a measure on how each variable contributes to the homogeneity in the resulting ML algorithm. The higher the value of mean decrease accuracy or mean decrease Gini score, the higher the importance of the variable in the model.

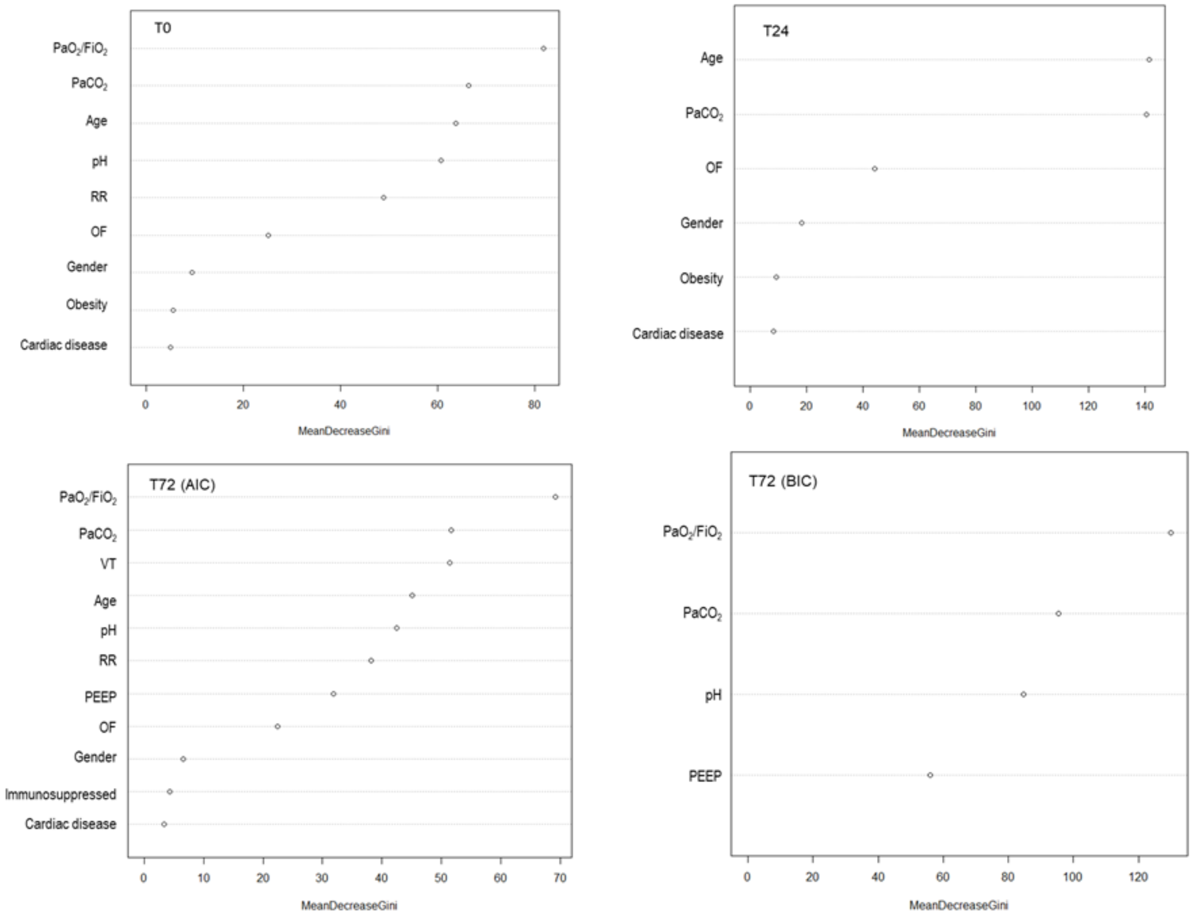

Figure S6. Calibration plots of the Multilayer Perceptron-based (A,B), Random Forest-based (C,D), Support Vector Machine-based (E,F), and logistic regression-based (G,H) MV >14 days prediction model at T72 for the 11- and 4-variable model, respectively. The intercept relates to calibration-in-the-large, which compares mean observed with mean predicted risks. The calibration slope reflects the coefficient of the calibration plot. The *c*-statistic indicates the discriminative ability.

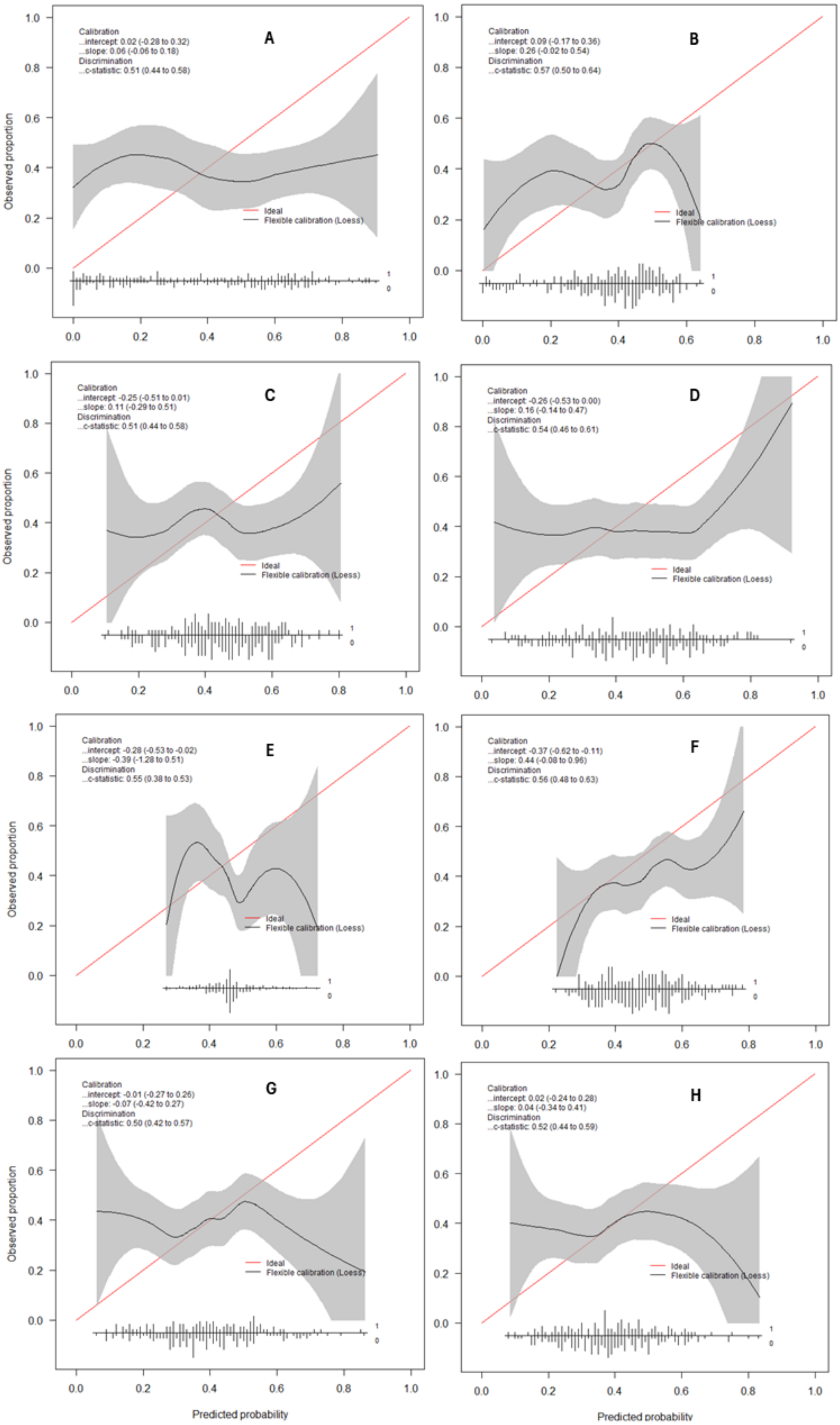

SUPPLEMENTARY REFERENCES

1. World Medical Association. World Medical Association Declaration of Helsinki: Ethical principles for medical research involving human subjects. JAMA 2013; 310:2191-2194.
2. Parsa-Parsi RW. The International Code of Medical Ethics of the World Medical Association. JAMA 2022; 328:2018-2021.
3. Collins GS, Reitsma JB, Altman DG, Mooms KGM. Transparent reporting of a multivariable prediction model for individual prognosis or diagnosis (TRIPOD): The TRIPOD statement. J Clin Epidemiol 2015; 68:112-121.
4. Villar J, Blanco J, Añón JM, Santos-Bouza A, Blanch L, Ambrós A, Gandía F, Carriedo D, Mosteiro F, Basaldúa S, et al. The ALIEN study: incidence and outcome of acute respiratory distress syndrome in the era of lung protective ventilation. Intensive Care Med 2011; 37:1932-1941.
5. Villar J, Pérez-Méndez L, Blanco J, Añón JM, Blanch L, Belda J, Santos-Bouza A, Fernández RL, Kacmarek RM; Spanish Initiative for Epidemiology, Stratification and Therapies for ARDS (SIESTA) network. A universal definition of ARDS: the PaO_2/FiO_2 ratio under s standard ventilatory setting – a prospective, multicenter validation study. Intensive Care Med 2013; 39:583-592.
6. Villar J, Blanco J. del Campo R, Andaluz-Ojeda D, Díaz-Domínguez FJ, Muriel A, Córcoles V, Suárez-Sipmann F, Tarancón C, González-Higueras E, et al; Spanish Initiative for Epidemiology, Stratification and Therapies for ARDS (SIESTA) network. Assessment of PaO_2/FiO_2 for stratification of patients with moderate and severe acute respiratory distress syndrome. BMJ Open 2015; 5:1006812.
7. Villar J, Mora-Ordoñez JM, Soler JA, Mosteiro F, Vidal A, Ambrós A, Fernández L, Murcia I, Civantos B, Romera MA, et al. The PANDORA study: prevalence and outcome of acute hypoxemic respiratory failure in the pre-COVID era. Crit Care Explor 2022; 4:e0684.
8. ARDS Definition Task Force; Ranieri VM, Rubenfeld GD, Thompson BT, Ferguson ND, Caldwell E, Fan E, Camporota L, Slutsky AS. Acute respiratory distress syndrome: The Berlin definition. JAMA 2012; 307:2526-2533.
9. Villar J, Ambrós A, Soler JA, Martínez D, Ferrando C, Solano R, Mosteiro F, Blanco J, Martín-Rodríguez C, Fernández MM, et al; Stratification and Outcome of Acute Respiratory Distress Syndrome (STANDARDS) network. Age, PaO_2/FiO_2 and plateau pressure score: a proposal for a

simple outcome score in patients with acute respiratory distress syndrome. Crit Care Med 2016; 44:1361-1369.

10. Villar J, Martín-Rodríguez C, Domínguez-Berrot AM, Fernández L, Ferrando C, Soler JA, Díaz-Lamas AM, González-Higueras E, Nogales L, Ambrós A; Spanish Initiative for Epidemiology, Stratification and Therapies for ARDS (SIESTA) Investigators Network. A quantile analysis of plateau and driving pressure: Effects on mortality in patients with acute respiratory distress syndrome receiving lung-protective ventilation. Crit Care Med 2017; 45:843-850.
11. Villar J, Ambrós A, Mosteiro F, Martínez D, Fernández L, Ferrando C, Carriedo D, Soler JA, Parrilla D, Hernández M; Spanish Initiative for Epidemiology, Stratification and Therapies for ARDS (SIESTA) network. A prognostic enrichment strategy for selection of patients with acute respiratory distress syndrome in clinical trials. Crit Care Med 2019; 47:377-385.
12. Vergouwe Y, Steyerberg EW, Eijkemans MJC, Habbeme JDF. Substantial effective sample sizes were required for external validation studies of predictive logistic regression models. J Clin Epidemiol 2005; 58:475-483.
13. Leisman DE, Harhay MO, Lederer DJ, Abramson M, Adjei AA, Bakker J, Ballas ZK, Barreiro E, Bell SC, Bellomo R, et al. Development and reporting of prediction models: Guidance for authors from editors of respiratory, sleep, and critical care journals. Crit Care Med 2020; 48:623-633.
14. Bernard GR, Artigas A, Brigham KL, Carlet J, Falke, Hudson L, Lamy M, Legall JR, Morris A, Spragg A. The American-European Consensus Conference on ARDS. Definitions, mechanisms, relevant outcomes, and clinical trial coordination. Am J Respir Crit Care Med 1994; 149:818-824.
15. Villar J, Pérez-Méndez L, López J, Belda J, Blanco J, Saralegui I, Suárez-Sipmann F, López J, Lubillo S, Kacmarek RM; HELP Network. An early PEEP/FiO2 trial identifies different degrees of lung injury in patients with acute respiratory distress syndrome. Am J Respir Crit Care Med 2007; 176:795-804.
16. Guerin C, Thompson T, Brower R. The ten diseases that look like ARDS. Intensive Care Med 2015; 41:1099-1102
17. Acute Respiratory Distress Syndrome Network. Ventilation with lower tidal volumes as compared with traditional tidal volumes for acute lung injury and the acute respiratory distress syndrome. N Engl J Med 2000; 342:1301-1308.

18. Kacmarek RM. Noninvasive respiratory support for postextubation respiratory failure. Respir Care 2019; 64:658-678.
19. Kacmarek RM, Villar J, Sulemanji D, Montiel R, Ferrando C, Blanco J, Koh Y, Soler JA, Martínez D, Hernández M, et al; Open Lung Approach Network. Open lung approach for the acute respiratory distress syndrome: A pilot, randomized controlled trial. Crit Care Med 2016; 44:32-42.
20. Villar J, González-Martín JM, Hernández-González J, Armengol MA, Fernández C, Martín-Rodríguez C, Mosteiro F, Martínez D, Sánchez-Ballesteros J, Ferrando C, et al. Predicting ICU mortality in acute respiratory distress syndrome patients using machine learning: the Predicting Outcome and STratifiCation of severity in ARDS (POSTCARDS) study. Crit Care Med 2023; 51:1638-1649.
21. Knaus WA, Draper EA, Wagner DP, Zimmerman JE. APACHE II. a severity of disease classification system. Crit Care Med 1985; 13:818-829.
22. Vincent JL, de Mendonça A, Cantraine F, Moreno R, Takala J, Suter PM, Sprung CL, Colardyn F, Blecher S. Use of the SOFA score to assess the incidence of organ dysfunction/failure in intensive care units: results of a multicenter, prospective study. Working group on "sepsis-related problems" of the European Society of Intensive Care Medicine. Crit Care Med 1998; 26:1793-1800.
23. Eke G, Bloos F, Wilson DC, Meybohm P; SepNet Critical Care Trials Group: Identification of developing multiple organ failure in sepsis patients with low or moderate SOFA scores. Crit Care 2018; 22:147.
24. Singer M, Deutschman CS, Seymour CW, Shankar-Hari M, Annane D, Bauer M, Bellomo R, Bernard GR, Chiche JD, Coopersmith CM, et al: The Third International Consensus Definition for Sepsis and Septic Shock. JAMA 2016; 315:801-810.
25. Levy MM, Fink MP, Marshall JC, Abraham E, Angus D, Cook D, Cohen J, Opal SM, Vincent JL, Ramsay G; SCCM/ESICM/ACCP/ATS(SIS. 2001 SCCM/ESICM/ACCP/ATS/SIS International Sepsis Definitions Conference. Crit Care Med 2003; 31:1250-1256.
26. Villar J, González-Martín JM, Añón JM, Ferrando C, Soler JA, Mosteiro F, Mora-Ordoñez JM, Ambrós A, Fernández L, Montiel R, et al. Clinical relevance of timing of assessment of ICU mortality in patients with moderate-to-severe acute respiratory distress syndrome. Sci Rep 2023; 13:1543.
27. Mandrekar JN. Receiver operating characteristic curve in diagnostic test assessment. J Thorac Oncol 2010; 5:1315-1316.

28. Villar J, González-Martín JM, Ambrós A, Mosteiro F, Martínez D, Fernández L, Soler JA, Parra L, Solano R, Soro M, et al; Spanish Initiative for Epidemiology, Stratification and Therapies of ARDS (SIESTA) network. Stratification for identification of prognostic categories in the acute respiratory distress syndrome (SPIRES) score. Crit Care Med 2021; 49:e920-e930.
29. Correlation matrix: what is it, how it works with examples. https://www.questionpro.com/blog/correlation-matrix/ (accessed on 15 November 2023).
30. Kim JH. Multicollinearity and misleading statistical results. Korean J J Anesthesiol 2019; 72:558-569.
31. Wang X, Meng L, Zhang J, Zhao Z, Zou L, Jia Z, Han X, Zhao L, Song M, Zong J, et al. Identification of ferroptosis-related molecular clusters and genes for diabetic osteoporosis based on the machine learning. Front Endocrinol 2023; 14:1189513.
32. What is Principal Component Analysis (PCA) and how it is used? https://www.sartorius.com/en/knowledge/science-snippets/what-is-principal-component-analysis-pca-and-how-it-is-used-507186#:~:text=Principal%20component%20analysis%2C%20or%20PCA,more%20easily%20visualized%20and%20analyzed (accessed on 15 November 2023).
33. Jolliffe IT, Cadima J. Principal components analysis: a review and recent developments. Philos Trans A Math Phys Eng Sci 2016; 374:20150202.
34. Gutierrez G. Artificial intelligence in the intensive care unit. Crit Care 2020; 24:101.
35. Scrucca L. GA: a package for genetic algorithms in R. J Statistical Software 2013; 53:1-37.
36. Escalona-Vargas D, Murphy P, Lowery CL, Eswaran H. Genetic algorithms for dipole location of fetal magnetocardiography. Ann Int Conf IEEE Eng Med Biol Soc 2016; 2016:904-907.
37. Vaderwater L, Brusic V, Wilson W, Macaulay L, Zhang P. An adaptive genetic algorithm for selection of blood-based biomarkers for prediction of Alzheimer´s disease progression. BMC Bioinformatics 2015; 16 Suppl 18 (Suppl 18): S1.
38. Gayou O, Das SK, Zhou SM, Marks LB, Parda DS, Miften M. A genetic algorithm for variable selection in logistic regression analysis of radiotherapy treatment outcomes. Med Phys 2008; 35:5426-5433.

39. González-Martin JM, Sánchez-Medina AJ, Alonso JB. Optimization of the prediction of financial problems in Spanish private health companies using genetic algorithms. Gac Sanit 2019; 33:462-467.

40. Vrieze SI. Model selection and psychological theory: a discussion of the differences between the Akaike information criterion (AIC) and the Bayesian information criterion (BIC). Psychol Methods 2012; 17:228-243.

41. Rashid M, Ramakrishnan M, Pulikkel V, Nandish S, Nair S, Shanbhag V, Thunga G. Artificial intelligence in acute respiratory distress syndrome: a systematic review. Artif Intell Med 2022; 131:102361.

42. Sayed M, Riaño D, Villar J. Predicting duration of mechanical ventilation in acute respiratory distress syndrome using supervised machine learning. J Clin Med 2021; 10:3824.

43. Boulesteix AL, Janitza S, Kruppa J, König IR. Overview of random forest methodology and practical guidance with emphasis on computational biology and bioinformatics. Wiley Interdiscip. Rev Data Min Knowl Discov 2012; 2:493–507.

44. Khalilzad Z, Hasasneh A, Tadj C. Newborn cry-based diagnostic system to distinguish between sepsis and respiratory distress syndrome using combined acoustic features. Diagnostics (Basel) 2022; 12:2802.

45. Jeon ET, Lee HJ, Park TY, Jin KN, Ryu B, Lee HW, Kim DH. Machine learning-based prediction of in-ICU mortality in pneumonia patients. Sci Rep 2023; 13:11527.

46. Royston P, Parmar MK, Sylvester R. Construction and validation of a prognostic model across several studies, with an application in superficial bladder cancer. Statist Med 2004; 23:907-926.

47. Steyerberg EW, Vergouwe Y. Towards better clinical prediction models: seven steps for development and an ABCD for validation. Eur Heart J 2014; 35:1925-1931.

48. Steyerberg EW, Harrel FE, Bosbsboom GJM, Eijkemans MJ, Vergouwe Y, Habbema JD. Internal validation of prediction models: efficiency of some procedures for logistic regression analysis. J Clin Epidemiol 2001; 54:774-781.

49. Saxena A, Mathur N, Pathak P, Tiwari P, Mathur SK. Machine learning model based on insulin resistance metagenes underpins genetic basis of type 2 diabetes. Biomolecules 2023; 13:432.

50. Ioannidis JPA. The proposal to lower P value thresholds to 0.005. JAMA 2018; 319:1429-1430.

51. Martínez-Taboada F, Redondo JI. The SIESTA (SEAAV Integrated evaluation sedation tool for anaesthesia) project: Initial development of a multifactorial sedation assessment tool for dogs. PLoS One 2020; 15: e0230799.
52. Van Calster B, Nieboer D, Vergouwe Y, De Cock B, Pencina MJ, Steyerberg EW. A calibration hierarchy for risk models was defined: from utopia to empirical data. J Clin Epidemiol 2016; 74:167-176.

APPENDIX I. Centers and Members of the PIONEER Project:

- Hospital Universitario Dr. Negrín, Las Palmas de Gran Canaria, Spain: Jesús Villar, Jesús M. González-Martín, Cristina Fernández, Estrella Gómez-Bentolila, Rosa L. Fernández, Pedro Rodríguez-Suárez;
- Hospital General Universitario, Ciudad Real, Spain: Alfonso Ambrós, Rafael del Campo, Carmen Martín-Rodríguez, Ana Bueno-González, Carmen Hornos-López;
- Complejo Hospitalario Universitario de La Coruña, La Coruña, Spain: Fernando Mosteiro, Lidia Pita-García, Ana M. Díaz-Lamas, Regina Arrojo;
- Hospital Universitario Virgen de Arrixaca, Murcia, Spain: Domingo Martínez, Juan A. Soler, Luís A. Conesa-Cayuela; Ana M. del Saz-Ortiz;
- Hospital General Universitario Rafael Méndez, Lorca, Murcia, Spain: Lucía Capilla;
- Hospital Universitario Río Hortega, Valladolid, Spain: Lorena Fernández, Jesús Sánchez-Ballesteros, Jesús Blanco, Arturo Muriel, Pablo Blanco-Schweizer, César Aldecoa, Jesús Rico-Feijoo, Alba Pérez, Silvia Martín-Alfonso;
- Complejo Hospitalario Universitario de León, León, Spain: Ana M. Domínguez, Francisco J. Díaz-Domínguez, Raúl I. González-Luengo, Demetrio Carriedo, Myriam González-Vaquero;
- Hospital Clínico Universitario, Valencia, Spain: Marina Soro, Javier Belda, Andrea Gutiérrez, Gerardo Aguilar;
- Hospital Clinic, Barcelona, Spain: Carlos Ferrando;
- Hospital Universitario La Paz, Madrid, Spain: José M. Añón, Belén Civantos, Mónica Hernández;
- Hospital Clínico Universitario, Valladolid, Spain: David Andaluz, Laura Parra, Leonor Nogales;
- Hospital Universitario Nuestra Señora de Candelaria, Tenerife, Spain: Raquel Montiel, Dácil Parrilla, Eduardo Peinado, Lina Pérez-Méndez;
- Hospital Virgen de la Luz, Cuenca, Spain: Rosario Solano, Elena González-Higueras;
- Hospital Fundación Jiménez Díaz, Madrid, Spain: Anxela Vidal, Denis Robaglia, César Pérez;
- Hospital Universitario Mutua Terrassa, Terrassa, Barcelona: M. Mar Fernández;
- Massachussets General Hospital, Boston, Massachusetts, USA: Robert M. Kacmarek (*deceased*).
- Li Ka Shing Knowledge Institute, St. Michael's Hospital, Toronto, Canada: Karen E.A. Burns.
- Aneurin Bevan University Health Board, Newport, Wales, United Kingdom: Tamas Szakmany;
- Leiden University Medical Center, Leiden, The Netherlands: Ewout W. Steyerberg;

APPENDIX 2. Members and Centers of the SIESTA Network:

- Jesús Villar, Rosa L. Fernández, Cristina Fernández, Jesús M. González-Martín, Pedro Rodríguez-Suárez, Estrella Gómez-Bentolila (Hospital Universitario Dr. Negrín, Las Palmas de Gran Canaria, Spain);
- Alfonso Ambrós, Rafael del Campo, Carmen Martín-Rodríguez, Ana Bueno-González, Carmen Hornos-López (Hospital General Universitario, Ciudad Real, Spain);
- Fernando Mosteiro, Ana M. Díaz-Lamas, Regina Arrojo, Lidia Pita-García (Complejo Hospitalario Universitario de La Coruña, La Coruña, Spain);
- Lorena Fernández, Jesús Sánchez-Ballesteros, Jesús Blanco, Arturo Muriel, Pablo Blanco-Schweizer, José Ángel de Ayala, César Aldecoa, Jesús Rico-Feijoo, Alba Pérez, Silvia Martín-Alfonso (Hospital Universitario Río Hortega, Valladolid, Spain);
- Domingo Martínez, Juan A. Soler, Ana M. del Saz-Ortiz, Luis A. Conesa-Cayuela (Hospital Universitario Virgen de Arrixaca, Murcia, Spain);
- Demetrio Carriedo, Ana M. Domínguez-Berrot, Francisco J. Díaz-Domínguez, Raúl I. González-Luengo, M. González-Vaquero (Complejo Hospitalario Universitario de León, León, Spain);
- Lucía Capilla (Hospital General Universitario Rafael Méndez, Lorca, Murcia, Spain);
- David Andaluz, Leonor Nogales, Laura Parra (Hospital Clínico Universitario, Valladolid, Spain);
- Elena González-Higueras, Rosario Solano, María J. Bruscas (Hospital Virgen de la Luz, Cuenca, Spain);
- Blanca Arocas, Marina Soro, Javier Belda, Andrea Gutiérrez, Ernesto Pastor, Gerardo Aguilar (Hospital Clínico Universitario, Valencia, Spain);
- Carlos Ferrando (Hospital Clinic, Barcelona, Spain);
- José M. Añón, Belén Civantos, Mónica Hernández (Hospital Universitario La Paz, Madrid, Spain);
- Raquel Montiel, Dácil Parrilla, Eduardo Peinado, Lina Pérez-Méndez (Hospital Universitario NS de Candelaria, Tenerife, Spain);
- Anxela Vidal, Denis Robaglia, César Pérez (Hospital Universitario Fundación Jiménez Díaz, Madrid, Spain);
- María del Mar Fernández (Hospital Universitario Mutua Terrassa, Terrassa, Barcelona, Spain);
- Eleuterio Merayo, Chanel Martínez-Jiménez, Ángeles de Celis-Álvarez (Hospital del Bierzo, Ponferrada, León, Spain);

- Juan M. Mora-Ordoñez, J. Francisco Martínez-Carmona, Álvaro Valverde-Monto, Victoria Olea-Jiménez (Hospital Regional Universitario de Málaga, Málaga, Spain);
- Concepción Tarancón, Silvia Cortés-Díaz (Hospital Virgen de la Concha, Zamora, Spain);
- Carmen Martín-Delgado (Hospital La Mancha Centro, Alcázar de San Juan, Ciudad Real, Spain);
- Francisca Prieto (Hospital Santa Bárbara, Puertollano, Ciudad Real, Spain);
- Isidro Prieto, Mario Chico, Darío Toral (Hospital Universitario 12 de Octubre, Madrid, Spain);
- Miguel A. Romera, Carlos Chamorro-Jambrina (Hospital Universitario Puerta de Hierro, Majadahonda, Madrid, Spain);
- Alec Tallet, Santiago Macías, Noelia Lázaro (Hospital General de Segovia, Segovia, Spain);
- Isabel Murcia, Ángel E. Pereyra (Hospital General Universitario de Albacete, Albacete, Spain);
- Francisco Alba, Ruth Corpas (Hospital NS del Prado, Talavera de la Reina, Toledo, Spain);
- David Pestaña, Pilar Cobeta, Adrián Mira (Hospital Universitario Ramón y Cajal, Madrid, Spain);
- Francisca Prieto (Hospital Santa Barbara, Puertollano, Ciudad Real, Spain);
- Lluis Blanch, Gemma Gomá, Gisela Pili (Corporació Sanitaria Parc Taulí, Sabadell, Barcelona, Spain);
- Antonio Santos-Bouza, Cristina Domínguez (Complejo Hospitalario Universitario de Santiago, Santiago de Compostela, La Coruña, Spain);
- Javier Collado, José I. Alonso (Hospital Río Carrión, Palencia, Spain);
- Alberto Indarte, María E. Perea (Hospital General Yagüe, Burgos, Spain);
- Ricardo Fernández, José I. Lozano (Hospital de Hellín, Albacete, Spain)
- Robert M. Kacmarek *(deceased)* (Massachussets General Hospital, Boston, Massachusetts, USA);
